# Gene gain and loss events in *Rickettsia *and *Orientia *species

**DOI:** 10.1186/1745-6150-6-6

**Published:** 2011-02-08

**Authors:** Kalliopi Georgiades, Vicky Merhej, Khalid El Karkouri, Didier Raoult, Pierre Pontarotti

**Affiliations:** 1 Unité de Recherche sur les Maladies Infectieuses et Tropicales Emergentes, URMITE, CNRS-IRD UMR 6236 IFR48 Faculté de Médecine, Université de la Méditerranée, Marseille, France; 2 Evolutionary biology and modeling, LATP UMR CNRS 6632 FR 3098 IFR48 University of Provence, Marseilles, France

## Abstract

**Background:**

Genome degradation is an ongoing process in all members of the *Rickettsiales *order, which makes these bacterial species an excellent model for studying reductive evolution through interspecies variation in genome size and gene content. In this study, we evaluated the degree to which gene loss shaped the content of some *Rickettsiales *genomes. We shed light on the role played by horizontal gene transfers in the genome evolution of *Rickettsiales*.

**Results:**

Our phylogenomic tree, based on whole-genome content, presented a topology distinct from that of the whole core gene concatenated phylogenetic tree, suggesting that the gene repertoires involved have different evolutionary histories. Indeed, we present evidence for 3 possible horizontal gene transfer events from various organisms to *Orientia *and 6 to *Rickettsia *spp., while we also identified 3 possible horizontal gene transfer events from *Rickettsia *and *Orientia *to other bacteria. We found 17 putative genes in *Rickettsia *spp. that are probably the result of *de novo *gene creation; 2 of these genes appear to be functional. On the basis of these results, we were able to reconstruct the gene repertoires of "proto-*Rickettsiales*" and "proto-*Rickettsiaceae*", which correspond to the ancestors of *Rickettsiales *and *Rickettsiaceae*, respectively. Finally, we found that 2,135 genes were lost during the evolution of the *Rickettsiaceae *to an intracellular lifestyle.

**Conclusions:**

Our phylogenetic analysis allowed us to track the gene gain and loss events occurring in bacterial genomes during their evolution from a free-living to an intracellular lifestyle. We have shown that the primary mechanism of evolution and specialization in strictly intracellular bacteria is gene loss. Despite the intracellular habitat, we found several horizontal gene transfers between *Rickettsiales *species and various prokaryotic, viral and eukaryotic species.

**Open peer review:**

Reviewed by Arcady Mushegian, Eugene V. Koonin and Patrick Forterre. For the full reviews please go to the Reviewers' comments section.

## Background

*Rickettsia *species are best known as the causative agents of vector-borne diseases, with rickettsial diseases representing an important cause of illness and death worldwide [1]. *Rickettsia *spp. have been isolated not only from hematophagous arthropod vectors, such as lice, ticks, fleas and mites [1], but also from several freshwater leeches [2-4], annelids, amoebae and plants [2]. The rickettsial species associated with arthropods are split into 2 major groups: the spotted fever group (SFG), which is associated with ticks, fleas and mites, and the typhus group (TG), which is smaller and is associated with human body lice (*Rickettsia prowazekii*) and fleas (*Rickettsia typhi*) [2,5]. *Rickettsia bellii *and *Rickettsia canadensis *exhibit important genomic divergences and branch outside both groups [5]. *Rickettsia *spp. have reduced genomes that vary in size from 1.1 MB for the TG, 1.2-1.4 MB for the SFG and 1.5 Mb for *R. bellii *[2]. Gene loss is thought to be a feature of the evolution of intracellular pathogenic bacteria [6-11]. In general, *Rickettsiales *genomes are still undergoing reduction, as supported by the *R. prowazekii *paradigm [12,13], which makes them an excellent model in which to study this process by observing interspecies variations in genome size and gene content [2]. The absence of a given gene in a genome (when compared to a related species) is often considered to be the result of its loss in that genome [11]. Consequently, the evolutionary process of gene loss in bacterial species has already been investigated in other studies [9,12,14,15].

Previous studies also showed that gene gain events occur in *Rickettsiales *[12,16] by duplication. Given the genetic isolation of *Rickettsiales*, alternative mechanisms for gene gain seemed impossible until the discovery of horizontal gene transfer (HGT) events in rickettsial genomes [9,17-20]. These findings made it acceptable to regard microbial genomes as dynamic entities that evolve by both losing and acquiring genes [21]. Therefore, when comparing closely related genomes, the relative absence of a given gene in one genome could potentially reflect gene gain in another genome.

Our objective was to study the evolution of *Rickettsia *and *Orientia *species from a free-living lifestyle to an intracellular one by considering gene loss and HGT events. For the first time, we compared 3 members of the intracellular *Rickettsiales *order (*Rickettsia *spp., *Orientia *spp., *Anaplasma *spp.) to a free-living, non-pathogenic alpha-proteobacterium (*Caulobacter *spp.). *Orientia tsutsugamushi *are obligate intracellular bacteria that live in mites and are the causative agent of scrub typhus [22], while *Anaplasma *spp. are pathogenic parasites that cause anaplasmosis in humans through tick bites [23]. As previously mentioned, gene loss in *Rickettsiales *has already been investigated, but here, we distinguished truly lost genes from genes gained by other species. Therefore, to identify HGT, we constructed systematic phylogenies for every gene that appeared to be missing. We were able to verify HGT events reported by previous studies, and we were able to describe previously unidentified events as well as to define the origin of each transferred gene. After identifying true gene loss and HGT, we reconstructed the ancestral gene sets of *alpha-proteobacteria*, proto-*Rickettsiales*, proto-*Rickettsiaceae *and proto-*Rickettsia*, which helped us to predict the number of genes lost during the passage from a free-living to an intracellular lifestyle (See Additional File 1: Figure S1). Finally, we attempted a reconstruction of the first *Rickettsia *genealogy of all gained genes.

## Results

We found that 701 riCOGs (COGs found only in *Rickettsia *spp.) were common to the 11 rickettsial species in our study, 995 riCOGs were absent from at least 1 of the 11 rickettsial species and 1,954 open reading frames (ORFs) were specific to 1 of the 11 species (See Additional File 1: Table S1). Functional characterization of the riCOGs is described in Figure S2 (See Additional File 1: Figure S2). We observed that 929 rioriCOGs (COGs obtained comparing *Rickettsia *spp. and *Orientia *spp.) were found in both *Rickettsia *and *Orientia*. In total, 173 rioriCOGs were found only in *Rickettsia*, and 119 were identified only in *Orientia*. Finally, cross-comparisons of *Rickettsia *spp., *Orientia *spp., *Anaplasma *spp. and *Caulobacter *detected 191 ricauCOGs (COGs obtained comparing all 4 genera) in all 4 genera, 102 in *Rickettsia*, *Orientia *and *Anaplasma *only, 281 in *Anaplasma *only and 1,982 in *Caulobacter *only.

### Phylogenomic analysis

**Figure 1:**
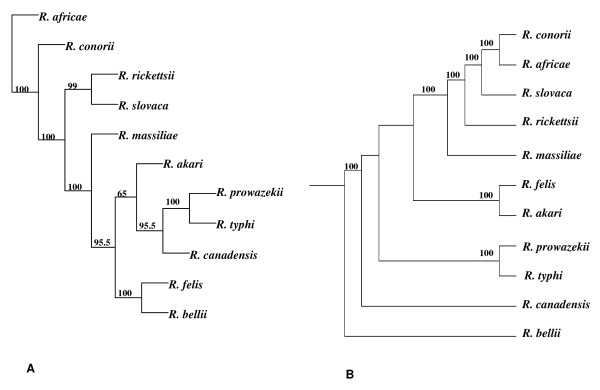
**Phylogenomic analysis of *Rickettsia *spp**. A. Gene content phylogeny constructed from the matrix of discrete characters using the neighbor-joining method. B. Whole core gene concatenated phylogenetic tree.

A phylogenomic tree constructed based on gene content (i.e., the presence or absence of COGs) showed a genus organization for *Rickettsiae *that was different from a whole core gene concatenated tree [24] (Figure 1) or even a phylogenetic tree based on 16S rRNA sequences [5]. On our phylogenomic tree, *R. felis *and *R. akari *are not placed within the SFG, in contrast to what would normally be expected. Rather, *R. felis *clusters with *R. bellii*, while *R. akari *clusters with the TG and *R. canadensis*. However, the branch leading to *R. akari *has a low bootstrap, probably due to phylogenetic uncertainty. This phylogenomic analysis shows that the rickettsial gene repertoires have different evolutionary histories. The phylogenomic tree suggests that differential gene loss and lateral gene acquisition may have played important roles in the evolution of some *Rickettsia *spp. Indeed, genes acquired from distant sources are more likely to supply novel traits that would set the recipient apart from its relatives [25]. We also analyzed the species organization for the different functional COG categories (See Additional File 1: Figure S3). Likewise, using the tree based on whole-genome content, phylogenomic analysis for the different functions revealed many topologies that differ from that of the whole core gene tree or the 16 S rRNA based tree. These results suggest that gene loss and HGT are relevant for all gene functions.

### HGT and phylogenetics

We used single-gene phylogenies to investigate the evolution of rickettsial gene content. When comparing 2 species, genes that are absent in one species could have either been lost in that species or gained in the other through HGT. To detect HGT, phylogenetic trees were constructed for *Rickettsiales *genes that were found to not be common to all *Rickettsiales *spp. and compared to the Tree of Life (See Material and Methods). Among the 995 riCOGs that were missing from at least 1 rickettsial species, we found 6 genes that were likely obtained by the other *Rickettsia *species *via *HGT (Table 1, See Additional File 2: Phylogenetic trees). These genes were acquired from several organisms, ranging from *gamma-proteobacteria *to eukaryotes. The functions of these transferred genes are diverse; the genes code for enzymes with synthase or proteolytic activities. Special interest should be given to the *metK *gene (riCOG00983), which codes for S-adenosylmethionine synthetase; phylogenies suggest that this gene was transferred from *gamma-proteobacteria *to *Rickettsia *species. Sequence analysis showed that the *metK *gene is degrading at different rates in the *Rickettsia *species that do not appear on the phylogenetic tree, while it is conserved in *R. akari*, and *R. felis*. This result agrees with the studies of Andersson [8] on the *metK *gene. This degradation confirms our hypothesis regarding gene loss in species where the gene is absent. Phylogenetic analysis showed that among the 129 rioriCOGs missing from at least one *Rickettsia *spp., 3 have likely been subject to HGT. These genes were obtained from different organisms, including *gamma*-*proteobacteria*, protists and viruses (Table 1). They code for enzymes with hydrolase activity and for ankyrin-repeat containing proteins.

**Table 1:** Genes gained by *Rickettsiales*.

riCOGs	donor species	annotation	biological process	gained by	lost by
riCOG01601	*γ -proteobacteria*	unknown	unknown	*R. canadensis*	

riCOG01580	*δ-proteobacteria*	unknown	unknown	*R. akari, R. felis*	

riCOG00373	*Bacteroidetes*	unknown	unknown	*R. canadensis*, SFG, *Orientia* Ikeda	*R. massiliae*

riCOG00835	*Cyanobacteria, Acidobacteria*	toxin of toxin antitoxin	toxin of toxin antitoxin	SFG	

riCOG00983	*γ-proteobacteria*	*met*K	S-adenosylmethionine synthesis	*R. akari, R. felis*, TG	*R. prowazekii*

riCOG01685	Eukaryotes	leucine-rich repeats	unknown	*R. felis, R. bellii*	

**rioriCOGs**					

rioriCOG00831	Canarypox virus	ankyrin-repeat containing protein	protein-protein interaction motif	*Orientia *spp.	

rioriCOG00862	*protists*	ankyrin-repeat containing protein	protein-protein interaction motif	*Orientia *spp.	

rioriCOG00673	*Cyanobacteria*	transposase & inactivated derivative	transposase activity	*Orientia *spp., R. endosymbiont of Ixodes scapularis	

Six riCOGs probably transferred by different bacteria, animals and plants to rickettsial species and 3 rioriCOGs probably transferred by *gamma-proteobacteria *species, viruses and protists to the *Orientia *genus. Transferred genes have various functions including protein-protein interaction and metabolic activity. HGT took place at different levels of evolution and at some cases it was followed by gene loss in some species.

### Rickettsiae as gene donors

We identified 3 examples where *Rickettsia *and *Orientia *were probably gene donors in HGT events. *Rickettsia *spp. from the SFG contributed a gene coding for a nucleotidyltransferase substrate binding protein to the *Firmicutes *spp. and *Bacteroidetes *spp., and the rickettsial ancestor contributed a gene coding for a putative permease to *gamma-proteobacteria *spp. *Orientia *species contributed a Na+/proline symporter histidine kinase gene to *Bacteroidetes *spp. These genes have various functions, including transposase and transferase activities (Table 2). We did not identify any HGT events in the genomes of *Anaplasma *spp., supporting the fact that none have ever been identified by any previous studies [10].

**Table 2:** *Rickettsiales *as gene donors.

	target species	function		gained from
**riCOGs**		**annotation**	**biological process**	

riCOG00139	*Firmicutes Bacteroidetes*	nucleotidyltransferase substrate binding	transferase activity	SFG

riCOG00530	*γ-proteobacteria*	putative permease	transmembrane transport	proto- *Rickettsiales*, other *alpha-proteobacteria*

**rioriCOGs**				

rioriCOG00847	*Bacteroidetes*	Na+/proline symporter histidine kinase	transposase activity	*Orientia *spp.

Two riCOGs and one rioriCOG probably transferred from *Rickettsia *and *Orientia *to *Firmicutes*, *gamma-proteobacteria *and *Bacteroidetes*. These genes code for transferase and transposases activity.

### Gene gain during rickettsial evolution

**Figure 2:**
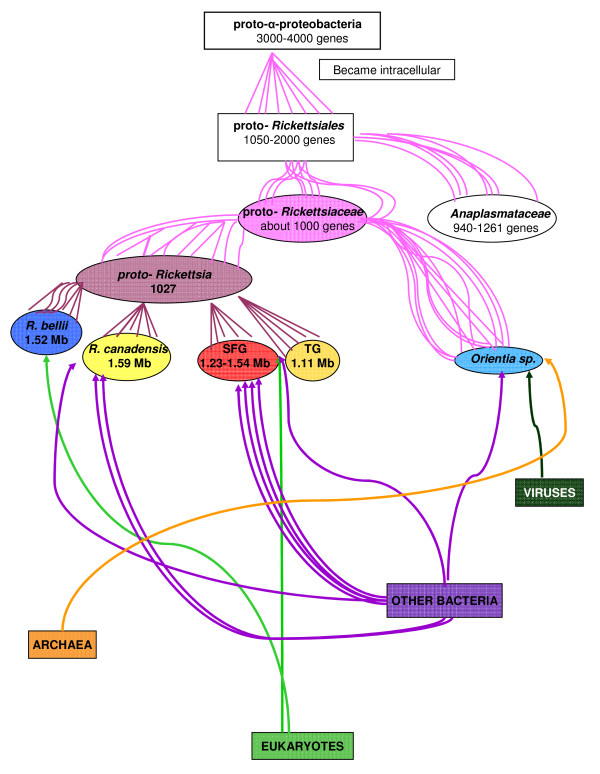
***Rickettsia *genealogy**. Probable gene gain events shaping the evolution of *Rickettsia*.

Gene gains occurred at different times during rickettsial evolution, and the rate of HGT varies among species (Figure 2). Genes were gained before the separation of the SFG and TG (2 genes) and, subsequently, during speciation events within the SFG (4 genes). We identified one HGT event in *Orientia tsutsugamushi *Ikeda but none in *Orientia tsutsugamushi *Boyrong. *R. canadensis*, with 3 horizontally transferred genes, exhibited the highest rate of HGT among the species studied. Most of the horizontally transferred genes in SFG were gained ancestrally; however, *R. akari *and *R. felis *gained 2 genes even after specialization. *R*. *prowazekii *and *R. typhi *are the only *Rickettsia *species in which no gene gains were found to have occurred after their separation from the SFG. In some species, HGT events were followed by gene loss (Table 1).

### De novo appearance

Genes present exclusively in *Rickettsia *spp. may correspond to genes that are the result of *de novo *gene formation. New genes often arise through the duplication of existing genes or through fusion/fission events [26]. Genes originating from non-coding DNA are extremely rare [26]. However, the short lengths of our 17 analyzed sequences indicate that they are likely to be newly arisen genes or, possibly, pseudogenes (Table 3). We only evaluated the 17 longest sequences because the probability that a sequence will be non-coding increases as sequence length decreases. Based on pair-wise ω ratio tests (ω = Ka/Ks = non synonymous/synonymous substitutions) on our 17 analyzed sequences, only 2 appear to be functional, given that their ω ratio is significantly less than 1. Of these genes, 3 seem to be evolving toward becoming coding regions in some lineages, while 12 others are apparently either non-functional or pseudogenes (their ω ratio is not significantly less than 1). The functions of the protein products of the true genes are unknown, and none have any annotated protein domains. Further studies are needed to investigate the functions of these novel genes.

**Table 3:** *De novo *genes

	Gene (sequence length *)	Found in	Size (nt)	Ka/Ks	P-value	Coding region	AT %
1	raf_ORF0703 (335 nt)	*R. conorii*	336	0.257	0.0357		57.6%
		*R. rickettsii*	477	1.628	0.046	Yes	

2	raf_ORF0265 (389 nt)	*R. conorii*	390	0.84	0.0233	Yes	49.6%

3	raf_ORF0391 (428 nt)	*R. felis*	351	1.5352	0.3244	No	51.1%

4	raf_ORF0649 (449 nt)	*R. massiliae*	309	1.355	0.1443		61.4%
		*R. felis*	363	1.794	0.7871		
		*R. bellii*	432	1.568	0.4576	No	

5	raf_ORF1011 (551 nt)	*R. felis*	555	1.239	0.9519	No	62.7%

6	raf_ORF1025 (311 nt)	*R. felis*	309	0.879	0.7758	No	63%

7	raf_ORF0586 (347 nt)	*R. conorii*	348	1.257	0.0719		58.2%
		*R. akari*	147	2.171	0.6816	No	

8	raf_ORF0724 (569 nt)	*R. rickettsii*	354	0.154	0.1146		58.8%
		*R bellii*	585	1.45	0.6896	No	

9	raf_ORF0390 (1001 nt)	*R. massiliae*	432	1.479	0.0888		55.3%
		*R. rickettsii*	579	0.639	0.1011		
		*R. felis*	744	0.649	0.7778		
		*R. bellii*	834	0.555	0.152	No	

10	raf_ORF0053 (488 nt)	*R. conorii*	474	0.615	0.1924		59%
		*R. massiliae*	489	0.579	0.2145		
		*R. rickettsii*	489	0.561	0.2427		
		*R. felis*	489	0.674	0.2498		
		*R. akari*	489	0.713	0.4433	No	

11	raf_ORF0348 (593 nt)	*R. conorii*	594	1.427	0.0494	Yes	58%
		*R. massiliae*	378	1.123	0.3122		
		*R. rickettsii*	405	0.492	0.0917		
		*R. felis*	603	1.517	0.9648		
		*R. akari*	576	1.076	0.3832	No	

12	raf_ORF0876 (378 nt)	*R. felis*	486	0.921	0.2213		51%
		*R. bellii*	486	1.354	0.636	No	

13	raf_ORF0172 (1169 nt)	*R. conorii*	1158	0.773	0.0564		52.8%
		*R. massiliae*	1170	0.764	0.0989		
		*R. rickettsii*	1185	0.069	0.2337	No	

14	raf_ORF0993 (320 nt)	*R. massiliae*	321	0.942	0.059		57%
		*R. typhi*	435	0.1905	0.544	No	

15	raf_ORF0046 (520 nt)	*R. conorii*	519	0.406	0.0174	Yes	58%
		*R. massiliae*	408	1.53	0.0684	No	
		*R. akari*	483	0.977	0.3574	No	

16	raf_ORF0275 (1394 nt)	*R. conorii*	1395	1.83	0.0262	Yes	55.6%
		*R. massiliae*	1404	1.084	0.0614	No	
		*R. rickettsii*	1395	0.767	0.0348	Yes	
		*R. felis*	1395	0.9	0.1505		
		*R. akari*	1422	0.873	0.2144		
		*R. canadensis*	1389	1.133	0.344	No	

17	raf_ORF0921 (647 nt)	*R. conorii*	576	0.62	0.0365	Yes	57.4%
		*R. felis*	645	0.642	0.2394	No	

When the ω ratio was significantly inferior to 1 (p-value < 0.05), we considered that we have a coding region. According to the ω ratio test, raf_ORF0703 and raf_ORF0265 are functional.

Their function in not known. On the contrary, the twelve following sequences seem to correspond to no coding regions, while raf_ORF0046, raf_ORF0275 and raf_ORF0921 seem to be non-functional. Only a transcriptomic analysis could show for sure if these regions are really coding or not. By chance, all of these genes are found in *R. africae *and to calculate the ω ratio we took as reference sequence the *R. africae *sequence.

* The length given in nucleotides is the sequence length in *Rickettsia africae *and the AT % content is the one corresponding to *Rickettsia africae*.

### Gene loss during rickettsial evolution

**Figure 3:**
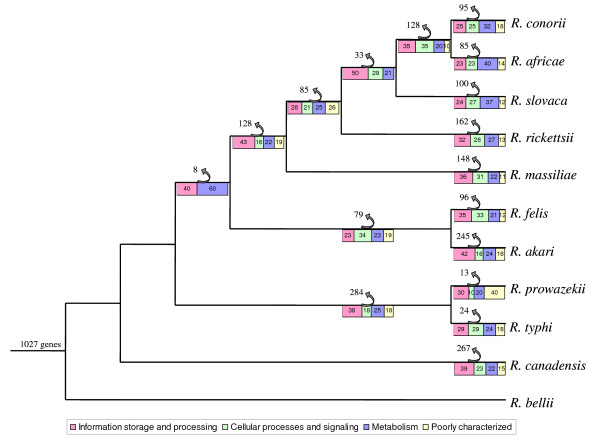
**Gene loss in *Rickettsia *spp**. The " proto-*Rickettsiae*" genome probably contained 1,027 genes. *R. bellii *showed no gene loss. The TG lost the largest number of genes, followed by *R. canadensis *and *R. akari*. The gene loss that occurred during the separation of the SFG from the TG mainly involves genes coding for metabolism and information storage and processing. Losses at species level equally affect genes of all functions. The figure represents the species tree as deduced from the concatenation of whole core gene of 11 *Rickettsia *spp. [Merhej *et al*., 2009 [24]]. Numbers at arrows show the number of lost genes. Genes were classified into four functional categories: information storage and processing in pink, cellular processes and signaling in green, metabolism in blue and poorly characterized functions in grey. Numbers in the colored squares indicate the percentage of lost genes in the corresponding category. Gain penalty was set at five using PARS algorithm.

After eliminating the genes gained by HGT, we were able to deduce the gene sets of "proto-*Rickettsia*" and "proto-*Rickettsiaceae*", which correspond to the ancestors of current rickettsial spp. and *Rickettsiaceae*, respectively, using the PARS algorithm [27] (See Material and Methods). A comparison of the gene content between species showed that *Rickettsia *spp. share 1,027 genes that were probably vertically transferred from " proto-*Rickettsia*" (Figure 3). *R. prowazekii *and *R. typhi *have lost the largest number of genes (284 genes), whereas *R. bellii *has maintained all of the 1,027 acquired genes. We found no gene loss in *R. bellii*, and indeed, this species has more genes than the rickettsial ancestor due to HGT. We only identified one such transfer because our study was restricted to the uncommon COGs. The group containing *R. conorii*, *R. massiliae*, *R. rickettsii *and *R. africae *lost many genes (128 genes) following its association with ticks and its separation from the *R. felis*/*R. akari *cluster (79 genes). Gene loss that occurred as the SFG separated from the TG mainly considered genes involved in metabolic functions and information storage and processing. Losses at the species level equally affect genes of all functions (Figure 3). The " proto- *Rickettsiaceae*" contained 1,944 genes. Thus, the *Rickettsiaceae *have lost 2,135 genes during their evolution to an intracellular lifestyle. Following their speciation and distinction from the *Orientia *genus, the *Rickettsiae *lost 1,015 genes (See Additional File 1: Figure S4).

## Discussion

Several studies have searched for HGT events in various species [28-35], including rickettsial species [18,19,36], but their results have rarely been used for genome reconstruction. In the instances where reconstructions have been performed, they have been based on assumptions and estimations by changing " gain and loss penalties" [31,32] that consider gene loss more likely to occur than gene gain. Furthermore, the origin of the transferred genes is not always clear [30]. Our phylogenetic analysis-based strategy allowed us to reconstruct, for the first time, the gene sets of " proto-*Rickettsiales*"," proto-*Rickettsiaceae*" and " proto- *alpha*-*proteobacteria*" by accounting for gene loss and gain events.

The content of the alpha-proteobacterial ancestor was estimated at 3,000-4,000 genes, which is also the result suggested in a previous study [15]. Looking further, we were able to determine that *Rickettsiaceae *lost 2,135 genes during their evolution to an intracellular lifestyle, and we described the rate of gene loss in 11 *Rickettsia *spp. We found that *R. bellii *has maintained all " proto-rickettsial" genes, while *R. prowazekii *and *R. typhi *have lost the largest number of ancestral genes and have the smallest genomes in *Rickettsia*. These 2 species present a restricted host range and exhibit extensive gene loss, together with reduced gene gain [9,36]. The differences in gene repertoires between current *Rickettsia *spp. are mainly the result of differential gene losses from the ancestor [9]. Gene loss at the species level is relevant for genes from all functional categories. The different functional repertoires seem to play important roles in the adaptation of *Rickettsia *spp. to their various hosts. Finally, the analysis of gene repertoires seems to be crucial for species definition [37]; differential gene losses help in the creation of new rickettsial species.

We found 9 genes that seem to have been gained by *Rickettsia *and *Orientia *spp. through HGT. Most gene gains took place before the separation of the SFG and the TG. However, horizontally acquired genes were then degraded [36], or completely lost, especially in the species belonging to the TG. Some genes seem to have been gained independently by different rickettsial species after their speciation. Transferred genes include transposases and ankyrin repeat-containing proteins and have various origins including different bacteria, animals and plants. Thus, we found genes encoding ankyrin repeat-containing proteins that appear to have been transferred from viruses and protists to *Orientia *species. These results suggest that genetic exchanges could have occurred between protists and their symbionts [38], as was found to be the case for *R. bellii *and amoebae [17]. Using a BLAST search against the " non-redundant" database (NR), we demonstrated that some of the horizontally transferred riCOGs that we identified show similarity to protist sequences, even when the probable donor species are not protists (See Additional File 1: Table S2). Protists, especially amoebae, appear to have played a significant role as a melting pot for genetic exchange [17,39]. The genome of *R. bellii *includes many genes related to those of amoebal symbionts [17], perhaps due to ancient gene exchanges between an ancestor of *R. bellii *and other amoebal hosts. It is plausible that the first host cell of *Rickettsia *was a protist [2]. Similarly, the intracellular lifestyles of *Rickettsia *and *Orientia *spp. allowed them to donate genes to *Firmicutes *spp., *Bacteroidetes *spp. and *gamma-proteobacteria *spp. The transferred genes encode for proteins with transposase, proteolysis and hydrolase activities. However, the biological significance of these HGT events cannot currently be inferred, and the biological impact of gene transfers remains to be investigated.

Phylogenies of *Rickettsia *spp. revealed that some gene sequences do not have any significant homologues in the NR database and may therefore have appeared *de novo*. The origin and function of such genes remain a mystery [40]. There are at least two steps involved in the evolution of new protein-coding genes from ancestral non-coding DNA. First, the DNA must be transcribed, and the locus that did not originally encode a protein has to acquire an ORF. Second, the new ORF must be transcribed through the use of a nearby existing gene promoter [26]. In *Rickettsia*, 15 of the 17 sequences analyzed are either nonfunctional or are in the process of adopting functionality. These sequences may also correspond to inactivated genes that are being degraded by mutation (pseudogenes). Of course, we should not neglect the fact that for closely related bacterial species, the Ka/Ks ratio might change over time because the selective consequences of non-synonymous change are not always effectively instantaneous [41]. It is also possible that these sequences may have been the result of HGT from lineages that have yet to be sequenced. It was recently proposed that such " ORFans" could also represent genes of viral or plasmidic origin [42,43]. Indeed, our candidates are short and AT-rich, as has been proposed for ORFans of viral origin (Table 3). However, given that they do not currently match any homologous sequences in any databases, we consider them to be probable *de novo *genes. It will therefore be interesting to determine the origin of these sequences and whether they are genuinely functional and, even more importantly, to determine their function.

## Conclusions

Previous studies have suggested that gene loss is a major evolutionary force that acts during the reductive evolution of intracellular bacteria [15,44,45]. Our phylogenetic analysis-based strategy of examining each missing gene allowed us to confirm that *Rickettsiales *are mainly shaped by gene loss. Genome size differences observed between obligate intracellular and free-living bacteria indeed result from genome reduction. However, we also detected HGT events that had not been previously identified and constructed the "proto-*Rickettsia*" genealogy based on quantitative genetic data. Therefore, our approach can be applied to better define bacterial evolutionary histories, and further cross-comparisons with other *Rickettsiales*, such as *Wolbachia *or *Pelagibacter ubique*, could help pinpoint specific HGT events leading to the intracellular lifestyle of *Rickettsia*. Finally, substantial horizontal gene transfers could help to accurately define bacterial spp. by allowing the phylogenetic history of genes that occur sporadically among multiple taxa to be traced.

## Methods

### Determination of COGs

Protein sets for 11 sequenced rickettsial species, 2 *Orientia tsutsugamushi *species, 3 *Caulobacter *species and 3 *Anaplasma *spp. were downloaded from NCBI [46]ftp://ftp.ncbi.nih.gov/genomes/Bacteria/. The following organisms were used in this study: *Rickettsia conorii *Malish 7 [NCBI: NC_003103]; *Rickettsia africae *ESF-5 [NCBI: NC_012633]; *Rickettsia massiliae *MTU5 [NCBI: NC_009900]; *Rickettsia rickettsii *Sheila Smith [NCBI: NC_009882]; *Rickettsia felis *URRWXCal2 [NCBI: NC_007109]; *Rickettsia akari *Hartford [NCBI: NC_009881]; *Rickettsia prowazekii *Madrid E [NCBI: NC_000963]; *Rickettsia typhi *Wilmington [NCBI: NC_006142]; *Rickettsia canadensis *McKiel [NCBI: NC_009879]; *Rickettsia bellii *RML369-C [NCBI: NC_007940]; *Orientia tsutsugamushi *Boyrong [NCBI: NC_009488]; *Orientia tsutsugamushi *Ikeda [NCBI: NC_010793]; *Caulobacter sp*. K31 [NCBI: NC_010338]; *Caulobacter crescentus *CB15 [NCBI: NC_002696]; *Caulobacter crescentus *NA1000 [NCBI: NC_011916]; *Anaplasma marginale *St. Maries [NCBI: NC_004842]; *Anaplasma marginale *Florida [NCBI: NC_012026]; and *Anaplasma phagocytophilum *[NCBI: NC_007797]. We also included the *Rickettsia slovaca *13-B proteome [project ID: 15712]. Orthologous genes were identified using the COGsoft program (E-value = 10^-20 ^and coverage ≥ 70%) [47].

### Gene content phylogeny

The COG data were used to construct a whole-genome phylogenetic tree based on gene content. We generated a matrix of binary discrete characters (" 0" and " 1" for absence and presence, respectively). Using this matrix, we constructed a phylogenetic tree implementing the neighbor-joining (NJ) method within PHYLIP (Phylogeny Inference Package) [48]. The intergenomic distance used was that defined by Snel [49] (Table 4).

**Table 4:** Gene content in the rickettsial genomes

*Rickettsia*	*conorii*	*slovaca*	*africae*	*massiliae*	*rickettsii*	*akari*	*felis*	*prowazekii*	*typhi*	*canadensis*	*bellii*
***conorii***	**1229**	1002	1025	850	911	785	826	758	760	752	805

***slovaca***	0.82	**1581**	1013	845	986	786	825	748	751	747	798

***africae***	0.83	0.82	**1242**	856	906	777	828	754	760	746	800

***massiliae***	0.69	0.64	0.69	**1330**	832	772	825	748	751	747	814

***rickettsii***	0.74	0.75	0.73	0.63	**1311**	782	811	740	743	742	783

***akari***	0.64	0.65	0.64	0.63	0.64	**1217**	792	743	747	740	770

***felis***	0.67	0.57	0.67	0.62	0.62	0.65	**1441**	774	775	758	887

***prowazekii***	0.87	0.86	0.87	0.86	0.85	0.86	0.89	**867**	784	724	750

***typhi***	0.92	0.91	0.92	0.91	0.90	0.90	0.94	0.95	**828**	727	752

***canadensis***	0.78	0.77	0.77	0.77	0.77	0.76	0.78	0.84	0.88	**969**	732

***bellii***	0.66	0.57	0.64	0.61	0.60	0.63	0.64	0.87	0.91	0.76	**1395**

The numbers of genes shared between genomes (upper right triangle), the percentage of genes shared between genomes (the total number divided by the number of genes in the smallest genome; lower left triangle) and the numbers of genes per genome (bold).

### Construction of phylogenies

We also conducted phylogenetic analyses of COGs, excluding those shared by all *Rickettsia *spp. Therefore, we analyzed the following COGs: 1) those found only in *Rickettsia *(riCOGs) and absent in at least one rickettsial species, 2) those obtained after comparisons of *Rickettsia *spp. and *Orientia *spp. (rioriCOGs) and 3) those obtained after comparisons of *Rickettsia *spp., *Orientia *spp., *Anaplasma *spp. and *Caulobacter *spp. (ricauCOGs). Homologous sequences were queried within an NR database, and multiple alignments of homologous sequences were made using MUSCLE [50]. Data producing a bias (such as skewing toward short sequences) or noise were automatically eliminated. Based on the multiple alignments, phylogenetic trees were constructed using three different methods: neighbor joining (NJ), maximum parsimony (MP) and maximum likelihood (ML). Paralogous sequences were detected by comparing gene trees against a reference species tree (Tree of Life), and functionality was verified using web databases including GeneOntology or NCBI's dbEST. These paralogous sequences were deleted from our study.

### Analysis of phylogenies

We used phylogenies to distinguish between gene loss and horizontal gene transfer. When the tree topology obtained from the phylogeny of a riCOG corresponded to the topology of the Tree of Life, the riCOG was considered to be lost by the species absent in the tree. In contrast, when the phylogeny presented *Rickettsiales *spp. anchored in a non-*alpha-proteobacteria *clade, we concluded that the riCOG was gained through HGT from the organism with which the rickettsial species was grouped. Due to the presence of many phylogenetic artifacts, we only considered the HGT hypothesis for trees with high bootstrap values (> 60) for at least one of the 3 methods. A TBLASTN was used for every possible HGT to eliminate the potential problem of missing data. Some phylogenies containing species only from *Rickettsiales *might be explained because the corresponding genes are short sequences that are inadequate for phylogenic analysis. These genes might be the result of putative *de novo *appearances. Thus, we estimated the pair-wise ω = Ka/Ks values for these genes and their BLASTN matches using the codeml software [51]. Sequences with ω < 1 likely represent a protein-coding exon. A χ^2^- test was used to determine whether ω ratios were significantly less than one. Therefore, when the *P *value was less than 0.05, the sequences were considered non-functional.

### Reconstruction of the ancestral gene set

After eliminating the possible gained genes, we reconstructed the gene set of " proto-*Rickettsiales*" and " proto-*Rickettsiaceae*" using a parsimony approach implemented in a two-pass algorithm [27]. The rickettsial species tree based on the whole gene core was coupled with the algorithm. The phyletic pattern of all COGs (i.e., the matrix indicating presence or absence) in each species analyzed was mapped onto this tree. The numbers of lost COGs were determined at ancestral nodes and at each leaf. We used the maximum possible gain penalty (i.e., 5) so that only gene losses appeared on the tree. *Caulobacter *spp. were used as outgroup for the reconstruction of the ancestral gene set.

## Abbreviations

SFG: spotted fever group; TG: typhus group; HGT: horizontal gene transfer; COG: cluster of orthologous genes; ORF: open reading frame; NR database: non-redundant database; riCOG: COG found only in *Rickettsia*; rioriCOG: COG obtained after comparison of *Rickettsia *spp. and *Orientia *spp.; ricauCOG: COG obtained after comparison of *Rickettsia *spp., *Orientia *spp., *Anaplasma *spp. and *Caulobacter *spp.

## Competing interests

The authors declare that they have no competing interests.

## Authors' contributions

PP and DR designed the research project. EK provided the COG data. KG and VM performed the genomic analysis. KG, VM, PP and DR analyzed the data. KG and VM wrote the paper. KG and VM contributed equally to the work. RD and PP revised the paper. All authors read and approved the final version.

## Reviewers' comments

### Reviewer's report 1

*Arcady Mushegian, Department of Bioinformatics, Stowers Institute for Medical Research, Kansas City, Missouri, USA*.

#### Reviewer 1

The manuscript by Georgiades *et al*. is concerned with evolution of gene content in *Rickettsiales*, a group of alpha-proteobacteria whose genomes must have experienced considerable gene losses in the process of becoming parasites. The authors used several existing algorithms to estimate specific gains and losses and to reconstruct a putative common ancestor of the group. The conclusions of the paper seem quite reasonable, but I have several concerns about methods and about specific examples shown in the Supplement 1. I suspect that a bit more detailed explanation would set this all right. More specifically, in order: Lines 123-124: "Genes that are absent in one species could either have been lost by this species or gained by another species *via *HGT" - This does not have to be an 'either-or' proposition, it could be both - has this been considered?

##### Author's response

*We agree with the reviewer on this point. An absent gene could have been gained and then lost, especially if the gain took place at an ancestral level. This idea has been considered; as shown in *Table 1, *we have mentioned the species that have gained genes by HGT and the species that have lost these genes after speciation (see also lines 166-167)*.

#### Reviewer 1

Line 177: "After eliminating the genes gained by HGT" - after eliminating genes gained by HGT, the authors could have rebuilt the gene content tree; have this been done? Is this "purged" gene-content tree closer to the 16S RNA tree than the initial gene-content tree? And throughout the rest of the paper, which 'species tree' of the three possible trees was used (it is sometimes indicated, but not always).

##### Author's response

*A gene content tree without the genes gained by HGT has not been rebuilt. We obtained strong evidence for only 12 HGT events. Therefore, the elimination of 12 genes from a phylogeny containing more than a thousand genes will not give a topology different from that of the initial tree*.

*Throughout the rest of the paper, the tree used for the reconstruction of the ancestral gene set was the whole core gene concatenated tree, while the single-gene phylogenies used for HGT identification were compared to the " Tree of Life", as defined by the 16S RNA sequences. This is now clarified in the paper (lines: 111-112, 132, 312-313, 318-319, 335-336)*.

#### Reviewer 1

Methods: line 284: In building neighbour-joining trees from the binary matrix of gene content, which intergenomic distance was used - the importance of the appropriate normalization has been emphasized in literature.

##### Author's response

*The intergenomic distance was that defined by Snel et al*. [49]*. The matrix of gene content was determined as follows: we calculated the percentage of genes shared between genomes i.e. the number of genes shared between genomes divided by the number of genes in the smallest genome (See *Table 4*)*.

#### Reviewer 1

"*Caulobacter *was used as outgroup" - in which cases? How is it compatible with the cases, discussed in the same paragraph, when *Rickettsiae *were attached to the non-alpha-proteobacteria?

##### Author's response

*Caulobacter was used as outgroup for the reconstruction of the ancestral gene set. The phrase was mistakenly placed in the wrong paragraph; this is now corrected (see Methods, lines 340-341)*.

#### Reviewer 1

Lines 305-307: " When the tree topology obtained from a riCOG was similar to the phylogeny of the tree from 16 S rRNA sequences..." - does 'similar' means 'same' here (i.e., the same when branches corresponding to the species lacking this COGs are pruned in the species tree), or 'similar but not the same'?

##### Author's response

*These lines have been rephrased in the paper (see Methods, lines 318-320). " Similar" here does not mean " identical"; it means " as close to the " Tree of Life" topology as possible"*.

#### Reviewer 1

Lines 307-309: " When, on the contrary, the phylogeny presented *Rickettsiales *spp. anchored in a non-*alpha proteobacteria *clade, we concluded that the riCOG was gained through HGT" - do the authors mean the whole riCOG, or only some genes in it? Table 1 seems to indicate the latter? What if there are no *alpha-proteobacteria *(other than *Rickettsia*) in the tree (see examples?)

##### Author's response

*We consider that a riCOG is a gene*.

*If there are no alpha-proteobacteria in a single-gene phylogeny (other than Rickettsia), then this is strong evidence of gene gain by Rickettsia only*.

#### Reviewer 1

Lines 324-325: " The whole genome sequence-based rickettsial species tree was coupled with the algorithm." - Which one (see above), and why the choice?

##### Author's response

*It is the whole core gene concatenated tree that was coupled to the algorithm. This point has been clarified in the Methods (lines 336-337). This tree gives a more robust and accurate phylogeny. Furthermore, this phylogeny corresponds to the phenotypic classification dividing Rickettsia spp. into the Typhus Group, Spotted Fever Group and a " group" of divergent species, R. bellii and R. canadensis *[5,24].

#### Reviewer 1

Sup. file 1: are trees in this file rooted or not? They seem to be unrooted but arbitrarily shown as rooted, which may cause visual artefacts, e.g.: riCOG01685: if the tree is rearranged properly (i.e., all Metazoans, including rotifer *Philodina*, shown as one clade which is well-supported), we see the *Rickettsia *clade as a deep branch between plants and animals; where is the HGT? rioriCOG00831: Canarypox virus and *Orientia *spp. sequences are intermingling branches without modest support - maybe both of them are basal, again no HGT? rioriCOG00862 may be a similar situation: looks like there is a *Trichomonas vaginalis *clade and an *Orientia *spp. clade - where is the HGT evidence?

##### Author's response

*According to the reviewer's comment, the trees were changed and are now rooted (*Additional File 2*)*.

*riCOG01685: Rickettsia found in a branch between plants and animals show a HGT from plants and animals to Rickettsia*.

*rioriCOG00831: Canarypox virus seems to surround Orientia spp.; it is more likely a HGT from Canarypox virus to Orientia spp*.

*rioriCOG00862: In this phylogeny, the only species present are Trichomonas vaginalis and Orientia species. This evidence supports a probable HGT toward Orientia spp*.

### Reviewer's report 2

*Eugene V. Koonin, NCBI, NLM, NIH, Bethesda, MD 20894, USA*.

#### Reviewer 2

Generally, a fine study, describing the evolutionary reconstruction of the evolution of a large group of bacterial endosymbionts. I think the study would benefit from employing a state of the art Maximum Likelihood method for the reconstruction of gene repertoires such as Count by Miklos Csuros (Csurös M. Count: evolutionary analysis of phylogenetic profiles with parsimony and likelihood. Bioinformatics. 2010 Aug 1; 26(15):1910-2). This is unlikely to change the conclusions dramatically but would yield more robust results.

##### Author's response

*We would like to thank the reviewer for the comment on the use of maximum likelihood method. However, we insist on our choice of the maximum parsimony method because the gene contents' evolution models are still unclear. Furthermore, we were able, using the PARS algorithm, to eliminate gained genes from the reconstruction and give high-gain penalties to make gene losses appear on the tree. We are, however, preparing a future approach using parsimony and branch lengths to reconstruct ancestral genomes (Royer-Carenzi M, Didier G, personal communication)*.

#### Reviewer 2

I am concerned about one conclusion of the paper, namely, that 17 genes in *Rickettsia *probably evolved *de novo*. The authors themselves consider alternatives such as rapid evolution and horizontal gene transfer from an unknown source, and I think these are actually much more plausible than the de novo explanation.

##### Author's response

*Rapid evolution and HGT from unknown sources are effectively plausible alternatives, as is a possible viral origin of these genes. These are scenarios that we are not neglecting. However, given that there is an important proportion of ORFans genes in bacterial genomes, it is not irrational to consider de novo creation. Finally, because they do not currently match with any homologs in any databases, we consider them to be probable de novo genes. This point has been reinforced in our Discussion (lines 256-261)*.

### Reviewer's report 3

*Patrick Forterre, Département de Microbiologie, Unité de Biologie Moléculaire du Gène chez les Extrêmophiles, Paris, France*.

#### Reviewer 3

Georgiades and co-workers report a phylogenomic analysis of the order *Rickettsiales*, in order to identify the mechanisms of reductive evolution that shape the history of this order. *Rickettsiales *are members of the phylum proteobacteria, subdivision alpha. They are intracellular parasites of eukaryotic cells and « closely » related to the ancestor of mitochondria. *Rickettsiales *are therefore interesting both from a medical and an evolutionary expertise. Several genomes of *Rickettsia *have been now sequenced and already submitted to extensive comparative genomic analyses because of their medical and evolutionary interest. The analysis of Georgiades and co-workers update previous analyses by including 11 genomes of *Rickettsiales *and a *Caulobacter *as outgroup. They have obtained from their phylogenomic analysis a more precise estimation of the gene content of the last common ancestors of *Rickettsia, Rickettsiales *and *Rickettsiae *Their result confirm that, as expected, intracellular *Rickettsia *have mainly evolved by gene loss.

The authors have focused on the distinction between gene loss and horizontal gene transfer, by analyzing individual phylogenies of genes absent in one species. They identify several cases of HGT and propose the direction of the transfer and potential sources for the donor (in the case of transfer to *Rickettsiae*) or the recipient (in the case of transfer from *Rickettsiae*). The number of transfer detected is very limited 12, *versus *more than 2000 gene losses), but raises interesting biological questions. In the case of transfer to *Rickettsiae*, the authors suggest that it involved either eukaryotes infected by a *Rickettsia *(possibly a protist) or another parasite (a virus or a bacterium) infecting the *Rickettsia *infected cell. They do not really discuss the case of transfer from *Rickettsiae*. They are very rare but quite interesting since they suggest that descendants of the parasitic bacterium that received the rickettsial gene within the infected eukaryotic cell were later on able to transfer this gene to free living bacterium. This confirms that intracellular pathogens are not completely close systems but can participate to the network of gene transfer in nature. For example, this suggests that a eukaryotic gene (or the gene of a eukaryotic virus) could be transferred to free living bacteria by the intermediate of an intracellular pathogenic bacteria.

##### Author's response

*HGT events both from and toward Rickettsiae are the result of their intracellular lifestyle. However, concerning the transfers from Rickettsiae, two out of three times, the donation of the gene took place at an ancestral level. It is the Spotted Fever Group and the proto-Rickettsiales that contributed genes and not the current Rickettsia spp. (See *Table 2*). This result suggests that the more specialized a species becomes, the more it becomes a closed system. This remark is even more convincing when looking at R. prowazekii, whose evolution is characterized exclusively by gene loss *[9,12]* (See lines 213-217). R. prowazekii is a true obligate intracellular human-specialized pathogen that does not participate in the network of gene transfer in nature*.

#### Reviewer 3

In their analysis, the authors compare the individual gene trees obtained to the 16 S rRNA tree to distinction between gene loss and horizontal gene transfer. It is unclear if this is the best strategy, since 16 S rRNA should be quite similar between *Rickettsiae*, with possibly a lack of resolution of the tree. They mention the whole core gene concatenated tree. Is this tree congruent with the 16 S rRNA tree? The authors could have updated this tree and use it as the reference species tree in their analysis. In any case, it should be interesting to have on the same figure the whole genome tree compared to the species tree (preferentially based on core gene phylogeny) (Figure 1).

##### Author's response

*The individual gene trees are compared to the " Tree of Life" phylogeny that corresponds to the classification of all species according to their 16S RNA sequences. We believe that this point is now clarified in the paper (lines 313-314, 318-320). The whole core gene concatenated tree is similar to the 16S RNA of rickettsial species but is not exactly identical (lines 112-113). According to the reviewer's comment on *Figure 1, *we presented both the whole genome phylogenomic tree and the whole core gene concatenated tree in the same figure (*Figure 1A, B*). We would like to thank the reviewer for this suggestion*.

#### Reviewer 3

The authors also identify a low number of new genes without homologues in data base (ORFans) in *Rickettsia*. They indicate that the origin and function of such genes remain a mystery. In fact, it has been recently shown that many ORFans in Archaeal and bacterial genomes have a viral or plasmidic origin (Cortez *et al*., 2009). These genes are usually short, AT-rich, and located in genome region with traces of active or ancient integrated extra chromosomal elements. What about the genes detected here? Is it possible to imagine a viral origin? What is known about plasmid and or viruses in *Rickettsiae*? Cortez *et al*., have described several putative provirus's (proplasmids) in several *Rickettsiae ***(Additional data file 3 in Cortez *et al*., 2009)**. It could be interesting to use their analysis to see if some ORFans genes or genes involved in loss and HGT described here belong to these putative integrated elements.

(Cortez D, Forterre P, Gribaldo S: **A hidden reservoir of integrative elements is the major source of recently acquired foreign genes and ORFans in Archaeal and bacterial genomes**. *Genome Biol*. 2009 **10(6):**R65).

##### Author's response

*Based on the reviewer's comment, except from the short length of our ORFans genes, which was already pointed out, the AT % content was verified. Indeed, these genes are AT- rich (See revised *Table 3*), so the possibility of a viral origin is now discussed in the paper (lines: 256-261)*.

## Supplementary Material

Additional file 1 **Four supplementary figures and two supplementary tables**. Figure S1. Phylogenetic analysis-based strategy. Figure S2. Functional characterization of the rickettsial COGs. The functional categories were determined by the COGs database http://www.ncbi.nlm.nih.gov/COG/grace/fiew.cgi. Figure S3. Phylogenomic clustering of *Rickettsia *spp. in the different COG functional categories. Topologies are different for each functional category. Figure S4. Gene loss. Tentative scenario of gene loss in *Rickettsiales *from " proto-*alpha-proteobacteria*" (a) to current *Rickettsia *spp. Gene sets of " proto-*alpha-proteobacteria*"," proto-*Rickettsiales*" (b) and " proto-*Rickettsiaceae*" (c) were estimated using the PARS algorithm with a gain penalty of five. Table S1. Determination of COGs. The total number of ORFs in the studied species, number of COGs and specific genes are indicated. Table S2. Horizontally transferred genes with sequences that produced a BLAST hit with protist sequences. E-value < e^-3 ^and identity >25%. Click here for file

Additional file 2 **Phylogenetic trees showing HGT events as generated by the ML method**. For genes gained by *Rickettsiales *the donors are colored in green and for the cases for which the *Rickettsiales *gave genes the receivers are colored in blue. Click here for file
