# Supplementary material for: Gene gain and loss events in Rickettsia and Orientia species
Source: Biol Direct. 2011 Feb 8;6:6. doi: 10.1186/1745-6150-6-6 (PMC3055210; doi:10.1186/1745-6150-6-6)
Supplement: Additional file 1 — Four supplementary figures and two supplementary tables. Figure S1. Phylogenetic analysis-based strategy. Figure S2. Functional characterization of the rickettsial COGs. The functional categories were determined by the COGs database http://www.ncbi.nlm.nih.gov/COG/grace/fiew.cgi. Figure S3. Phylogenomic clustering of Rickettsia spp. in the different COG functional categories. Topologies are different for each functional category. Figure S4. Gene loss. Tentative scenario of gene loss in Rickettsiales from " proto-alpha-proteobacteria" (a) to current Rickettsia spp. Gene sets of " proto-alpha-proteobacteria"," proto-Rickettsiales" (b) and " proto-Rickettsiaceae" (c) were estimated using the PARS algorithm with a gain penalty of five. Table S1. Determination of COGs. The total number of ORFs in the studied species, number of COGs and specific genes are indicated. Table S2. Horizontally transferred genes with sequences that produced a BLAST hit with protist sequences. E-value < e-3 and identity >25%. [file 1745-6150-6-6-S1.PDF]

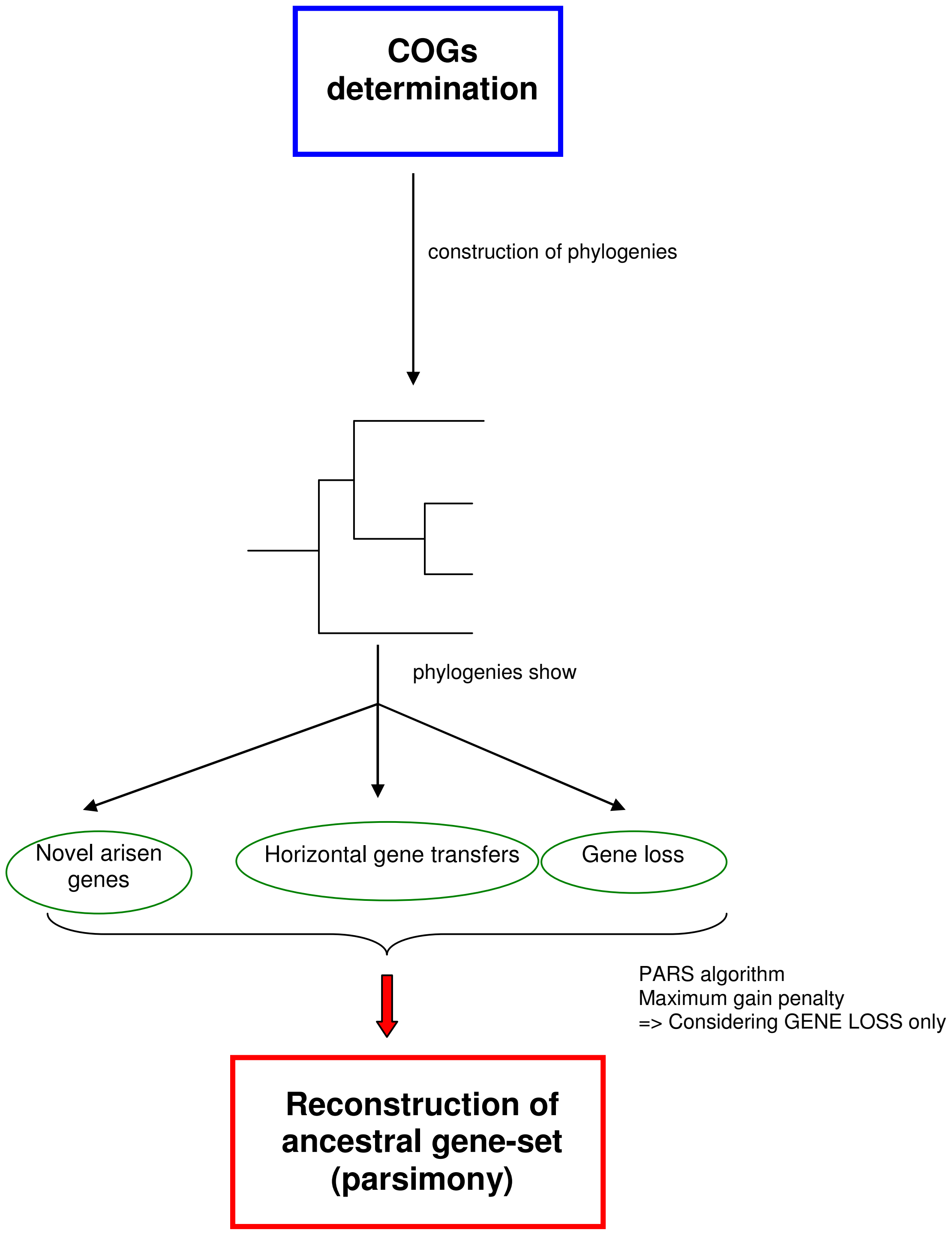
COGs determination
construction of phylogenies
phylogenies show
Novel arisen genes
Horizontal gene transfers
Gene loss
PARS algorithm
Maximum gain penalty
=> Considering GENE LOSS only
Reconstruction of ancestral gene-set (parsimony)

Figure S1

	Total ORFs	Genes in riCOGs	Specific genes	Percentage of genes that have at least one orthologous in Rickettsia spp.
Rickettsia conorii	1229	1192	37	96.99
Rickettsia africae	1242	1201	41	96.70
Rickettsia slovaca	1581	1283	298	81.15
Rickettsia rickettsii	1311	1160	151	88.48
Rickettsia massiliae	1330	1086	244	81.65
Rickettsia akari	1217	942	275	77.40
Rickettsia felis	1441	1093	348	75.85
Rickettsia prowazekii	867	823	44	94.93
Rickettsia typhi	828	811	17	97.95
Rickettsia canadensis	969	822	147	84.83
Rickettsia bellii	1395	1043	352	74.77
	**Total ORFs**	**Genes in rioriCOGs/ricauCOGs**	**Specific genes**	**Percentage of *Orientia* genes that have at least one orthologous in *Rickettsia* spp. rioriCOGs/ricauCOGs**
Orientia Boyrong	1149	684/394	465/755	59.53/34.29
Orientia Ikeda	1932	684/394	1248/1538	35.40/20.39
	**Total ORFs**	**Genes in ricauCOGs**	**Specific genes**	**Percentage of *Anaplasma* and *Caulobacter* genes that have at least one orthologous in *Rickettsia* spp. ricauCOGs**
Anaplasma marginale Florida	938	249	689	26.54
Anaplasma marginale St. Marie	1071	249	822	23.24
Anaplasma phagocytophilum	1511	249	1262	16.47
Caulobacter sp. K31	5437	2315	3122	42.57
Caulobacter crescentus CB15	3713	2315	1398	62.34
Caulobacter crescentus NA1000	3875	2315	1560	59.74

Table S1

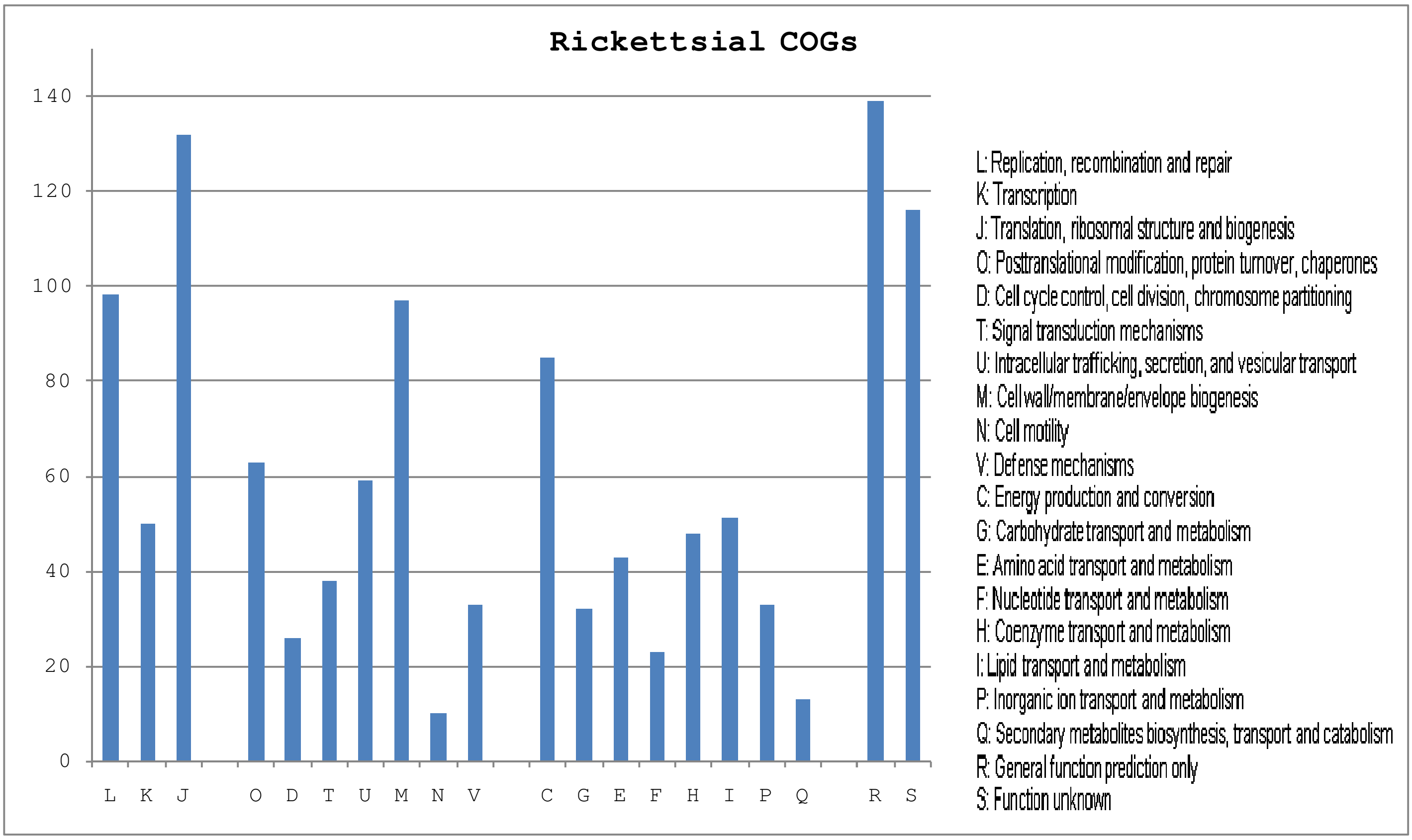
Rickettsial COGs
140
120
100
80
60
40
20
0
L K J O D T U M N V C G E F H I P Q R S
L: Replication, recombination and repair
K: Transcription
J: Translation, ribosomal structure and biogenesis
O: Posttranslational modification, protein turnover, chaperones
D: Cell cycle control, cell division, chromosome partitioning
T: Signal transduction mechanisms
U: Intracellular trafficking, secretion, and vesicular transport
M: Cell wall/membrane/envelope biogenesis
N: Cell motility
V: Defense mechanisms
C: Energy production and conversion
G: Carbohydrate transport and metabolism
E: Amino acid transport and metabolism
F: Nucleotide transport and metabolism
H: Coenzyme transport and metabolism
I: Lipid transport and metabolism
P: Inorganic ion transport and metabolism
Q: Secondary metabolites biosynthesis, transport and catabolism
R: General function prediction only
S: Function unknown

Figure S2

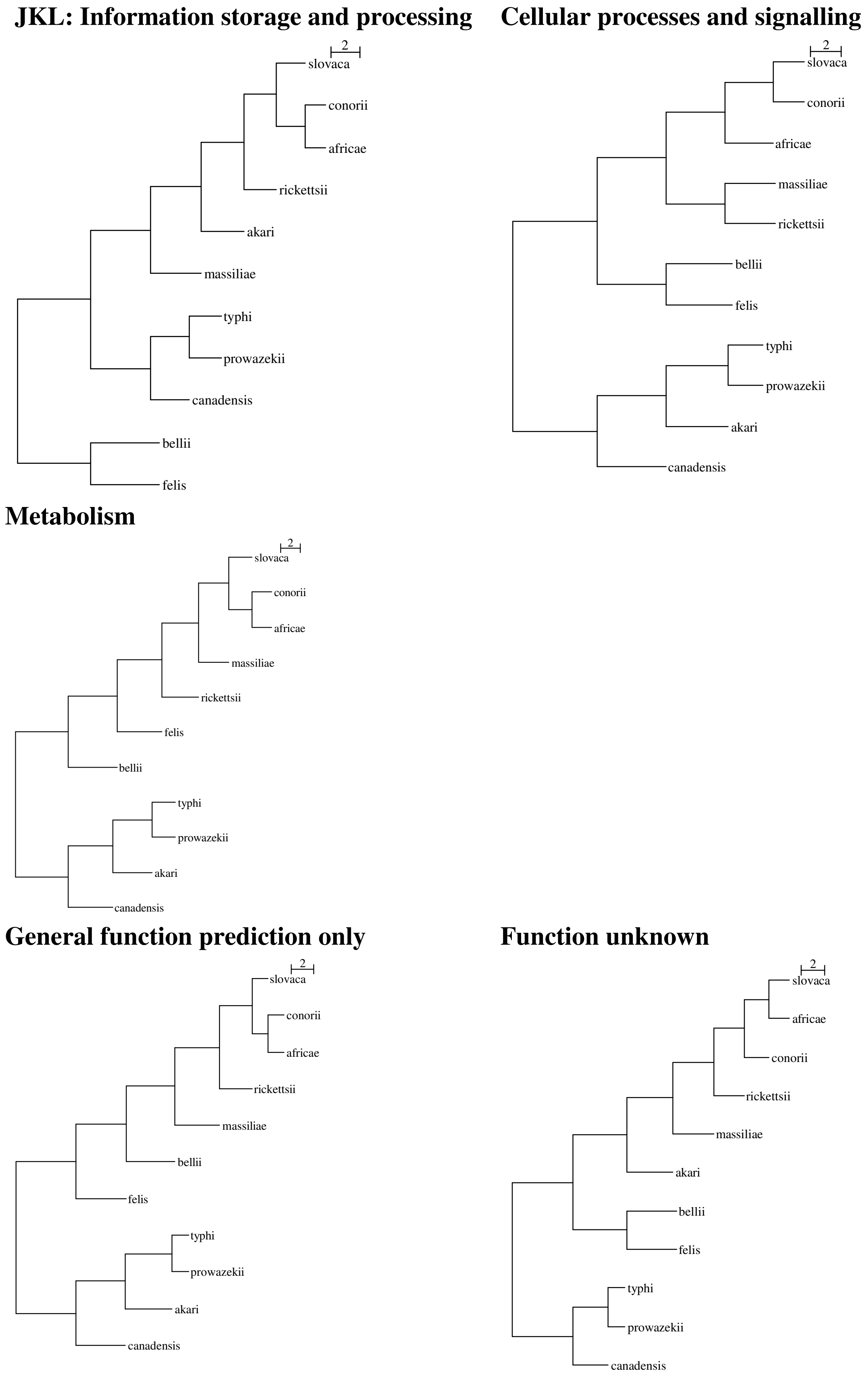
JKL: Information storage and processing
2
slovaca
conorii
africae
rickettsii
akari
massiliae
typhi
prowazekii
canadensis
bellii
felis
Cellular processes and signalling
2
slovaca
conorii
africae
massiliae
rickettsii
bellii
felis
typhi
prowazekii
akari
canadensis
Metabolism
2
slovaca
conorii
africae
massiliae
rickettsii
felis
bellii
typhi
prowazekii
akari
canadensis
General function prediction only
2
slovaca
conorii
africae
rickettsii
massiliae
bellii
felis
typhi
prowazekii
akari
canadensis
Function unknown
2
slovaca
africae
conorii
rickettsii
massiliae
akari
bellii
felis
typhi
prowazekii
canadensis

Figure S3

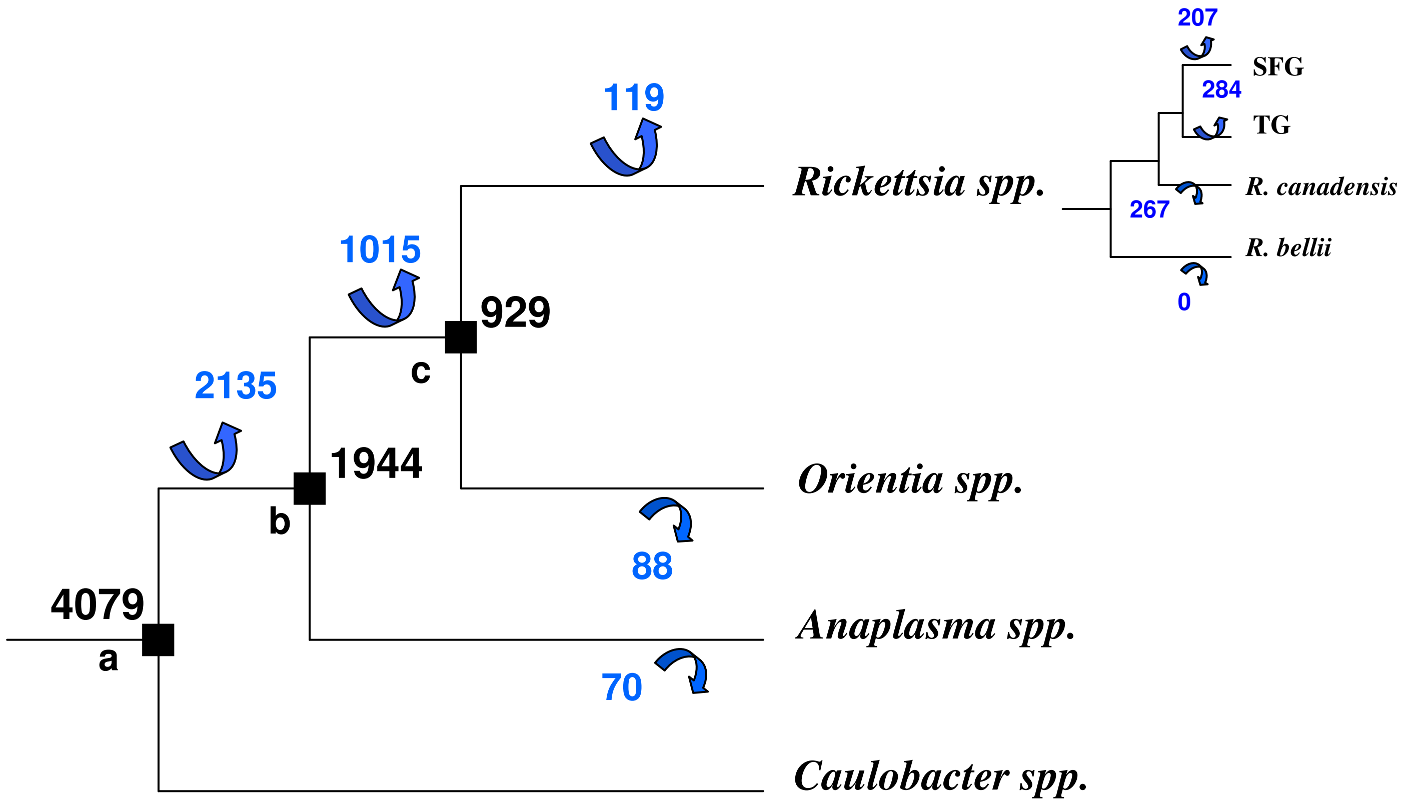
207
SFG
284
TG
267
R. canadensis
R. bellii
0
119
Rickettsia spp.
1015
929
c
2135
1944
b
Orientia spp.
88
4079
a
Anaplasma spp.
70
Caulobacter spp.

Figure S4

riCOGs/rioriCOGs	Protists	e-value	% identity
riCOG00520	*Dictyostelium discoideum*	$7.29e^{-27}$	34.36
	Thalassiosira pseudonana	$1.08e^{-114}$	41.44
riCOG00983	*Trichomonas vaginalis*	$1.16e^{-9}$	67.44
riCOG01685	*Dictyostelium discoideum*	$1.54e^{-8}$	28.80
	Tetrahymena thermophila	$7.2e^{-5}$	27.91
	Paramecium tetraurelia	$7.4e^{-5}$	29.23
	Trypanosoma cruzi	$7.8e^{-8}$	33
	Trypanosoma brucei	$1e^{-3}$	32.13
	Leishmania major	$1.4e^{-3}$	36.73
	Leishmania infantum	$6.7e^{-3}$	35
	Trichomonas vaginalis	$5.6e^{-5}$	42.68
	Phaeodactylum tricomutum	$8.4e^{-3}$	41.77
	Thalassiosira pseudonana	$2.4e^{-6}$	40.74

Table S2
