# Supplementary material for: Gene gain and loss events in Rickettsia and Orientia species
Source: Biol Direct. 2011 Feb 8;6:6. doi: 10.1186/1745-6150-6-6 (PMC3055210; doi:10.1186/1745-6150-6-6)
Supplement: Additional file 2 — Phylogenetic trees showing HGT events as generated by the ML method. For genes gained by Rickettsiales the donors are colored in green and for the cases for which the Rickettsiales gave genes the receivers are colored in blue. [file 1745-6150-6-6-S2.PDF]

Gene gain in Rickettsiales

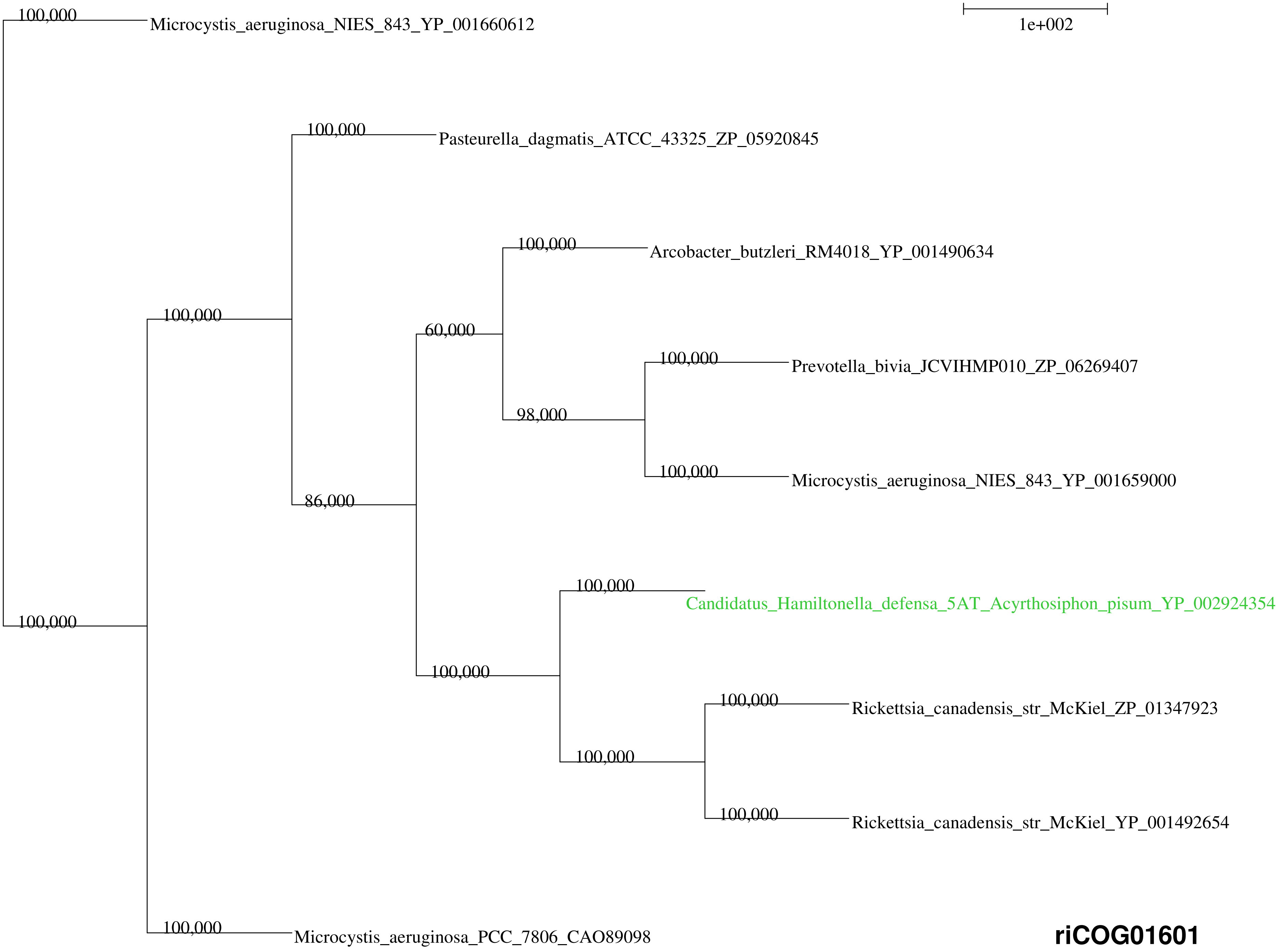
1e+002
Microcystis_aeruginosa_NIES_843_YP_001660612
Pasteurella_dagmatis_ATCC_43325_ZP_05920845
Arcobacter_butzleri_RM4018_YP_001490634
Prevotella_bivia_JCVIHMP010_ZP_06269407
Microcystis_aeruginosa_NIES_843_YP_001659000
Candidatus_Hamiltonella_defensa_5AT_Acyrthosiphon_pisum_YP_002924354
Rickettsia_canadensis_str_McKiel_ZP_01347923
Rickettsia_canadensis_str_McKiel_YP_001492654
Microcystis_aeruginosa_PCC_7806_CAO89098
100,000
60,000
98,000
86,000

riCOG01601

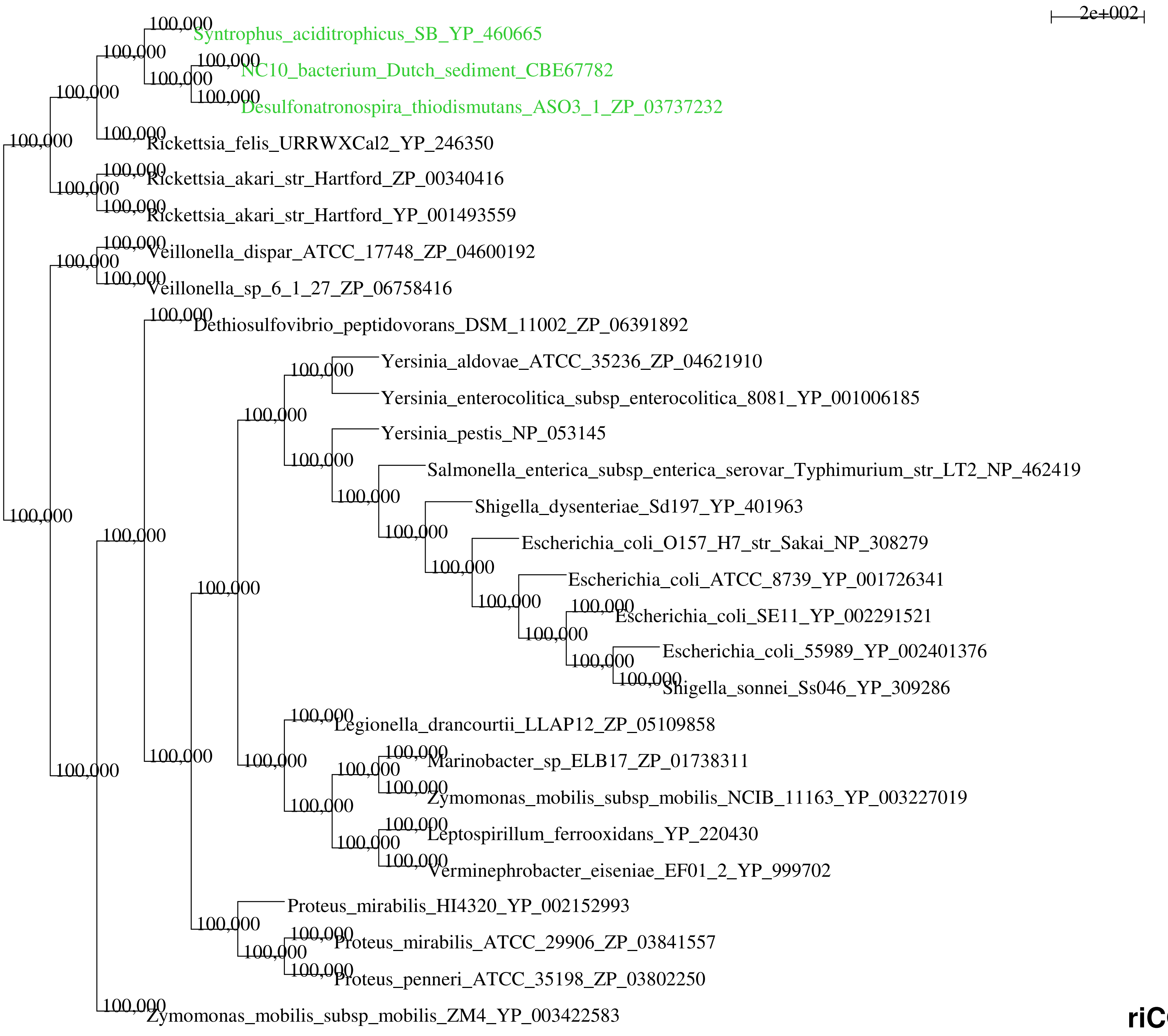
2e+002
Syntrophus_aciditrophicus_SB_YP_460665
NC10_bacterium_Dutch_sediment_CBE67782
Desulfonatronospira_thiodismutans_ASO3_1_ZP_03737232
Rickettsia_felis_URRWXCal2_YP_246350
Rickettsia_akari_str_Hartford_ZP_00340416
Rickettsia_akari_str_Hartford_YP_001493559
Veillonella_dispar_ATCC_17748_ZP_04600192
Veillonella_sp_6_1_27_ZP_06758416
Dethiosulfovibrio_peptidovorans_DSM_11002_ZP_06391892
Yersinia_aldovae_ATCC_35236_ZP_04621910
Yersinia_enterocolitica_subsp_enterocolitica_8081_YP_001006185
Yersinia_pestis_NP_053145
Salmonella_enterica_subsp_enterica_serovar_Typhimurium_str_LT2_NP_462419
Shigella_dysenteriae_Sd197_YP_401963
Escherichia_coli_O157_H7_str_Sakai_NP_308279
Escherichia_coli_ATCC_8739_YP_001726341
Escherichia_coli_SE11_YP_002291521
Escherichia_coli_55989_YP_002401376
Shigella_sonnei_Ss046_YP_309286
Legionella_drancourtii_LLAP12_ZP_05109858
Marinobacter_sp_ELB17_ZP_01738311
Zymomonas_mobilis_subsp_mobilis_NCIB_11163_YP_003227019
Leptospirillum_ferrooxidans_YP_220430
Verminephrobacter_eiseniae_EF01_2_YP_999702
Proteus_mirabilis_HI4320_YP_002152993
Proteus_mirabilis_ATCC_29906_ZP_03841557
Proteus_penneri_ATCC_35198_ZP_03802250
Zymomonas_mobilis_subsp_mobilis_ZM4_YP_003422583
100,000

riCOG01580

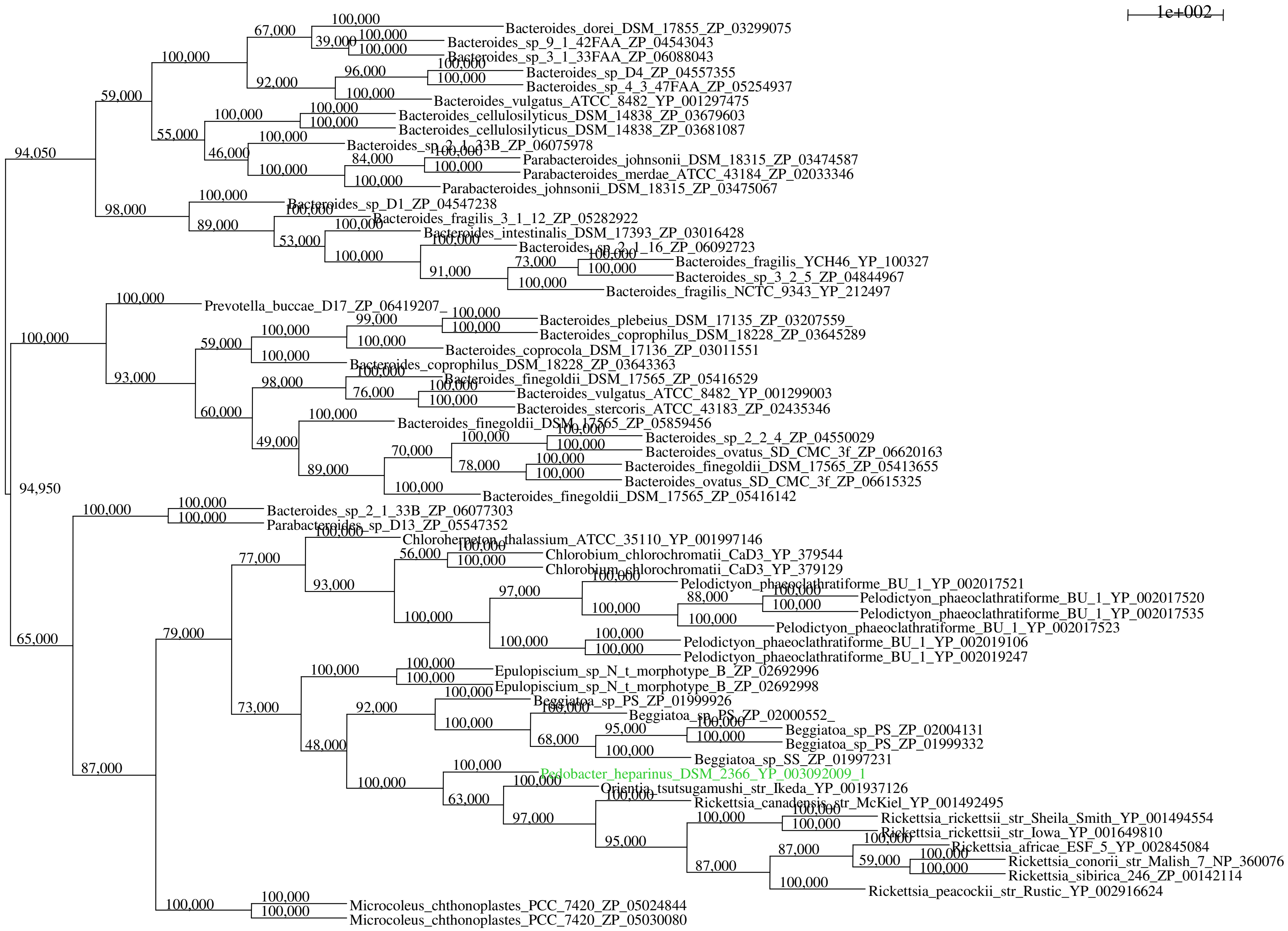
1e+002
Bacteroides_dorei_DSM_17855_ZP_03299075
Bacteroides_sp_9_1_42FAA_ZP_04543043
Bacteroides_sp_3_1_33FAA_ZP_06088043
Bacteroides_sp_D4_ZP_04557355
Bacteroides_sp_4_3_47FAA_ZP_05254937
Bacteroides_vulgatus_ATCC_8482_YP_001297475
Bacteroides_cellulosilyticus_DSM_14838_ZP_03679603
Bacteroides_cellulosilyticus_DSM_14838_ZP_03681087
Bacteroides_sp_2_1_33B_ZP_06075978
Parabacteroides_johnsonii_DSM_18315_ZP_03474587
Parabacteroides_merdae_ATCC_43184_ZP_02033346
Parabacteroides_johnsonii_DSM_18315_ZP_03475067
Bacteroides_sp_D1_ZP_04547238
Bacteroides_fragilis_3_1_12_ZP_05282922
Bacteroides_intestinalis_DSM_17393_ZP_03016428
Bacteroides_sp_2_1_16_ZP_06092723
Bacteroides_fragilis_YCH46_YP_100327
Bacteroides_sp_3_2_5_ZP_04844967
Bacteroides_fragilis_NCTC_9343_YP_212497
Prevotella_buccae_D17_ZP_06419207_
Bacteroides_plebeius_DSM_17135_ZP_03207559_
Bacteroides_coprophilus_DSM_18228_ZP_03645289
Bacteroides_coprocola_DSM_17136_ZP_03011551
Bacteroides_coprophilus_DSM_18228_ZP_03643363
Bacteroides_finegoldii_DSM_17565_ZP_05416529
Bacteroides_vulgatus_ATCC_8482_YP_001299003
Bacteroides_stercoris_ATCC_43183_ZP_02435346
Bacteroides_finegoldii_DSM_17565_ZP_05859456
Bacteroides_sp_2_2_4_ZP_04550029
Bacteroides_ovatus_SD_CMC_3f_ZP_06620163
Bacteroides_finegoldii_DSM_17565_ZP_05413655
Bacteroides_ovatus_SD_CMC_3f_ZP_06615325
Bacteroides_finegoldii_DSM_17565_ZP_05416142
Bacteroides_sp_2_1_33B_ZP_06077303
Parabacteroides_sp_D13_ZP_05547352
Chloroherpeton_thalassium_ATCC_35110_YP_001997146
Chlorobium_chlorochromatii_CaD3_YP_379544
Chlorobium_chlorochromatii_CaD3_YP_379129
Pelodictyon_phaeoclathratiforme_BU_1_YP_002017521
Pelodictyon_phaeoclathratiforme_BU_1_YP_002017520
Pelodictyon_phaeoclathratiforme_BU_1_YP_002017535
Pelodictyon_phaeoclathratiforme_BU_1_YP_002017523
Pelodictyon_phaeoclathratiforme_BU_1_YP_002019106
Pelodictyon_phaeoclathratiforme_BU_1_YP_002019247
Epulopiscium_sp_N_t_morphotype_B_ZP_02692996
Epulopiscium_sp_N_t_morphotype_B_ZP_02692998
Beggiatoa_sp_PS_ZP_01999926
Beggiatoa_sp_PS_ZP_02000552_
Beggiatoa_sp_PS_ZP_02004131
Beggiatoa_sp_PS_ZP_01999332
Beggiatoa_sp_SS_ZP_01997231
Pedobacter_heparinus_DSM_2366_YP_003092009_1
Orientia_tsutsugamushi_str_Ikeda_YP_001937126
Rickettsia_canadensis_str_McKiel_YP_001492495
Rickettsia_rickettsii_str_Sheila_Smith_YP_001494554
Rickettsia_rickettsii_str_Iowa_YP_001649810
Rickettsia_africae_ESF_5_YP_002845084
Rickettsia_conorii_str_Malish_7_NP_360076
Rickettsia_sibirica_246_ZP_00142114
Rickettsia_peacockii_str_Rustic_YP_002916624
Microcoleus_chthonoplastes_PCC_7420_ZP_05024844
Microcoleus_chthonoplastes_PCC_7420_ZP_05030080

riCOG00373

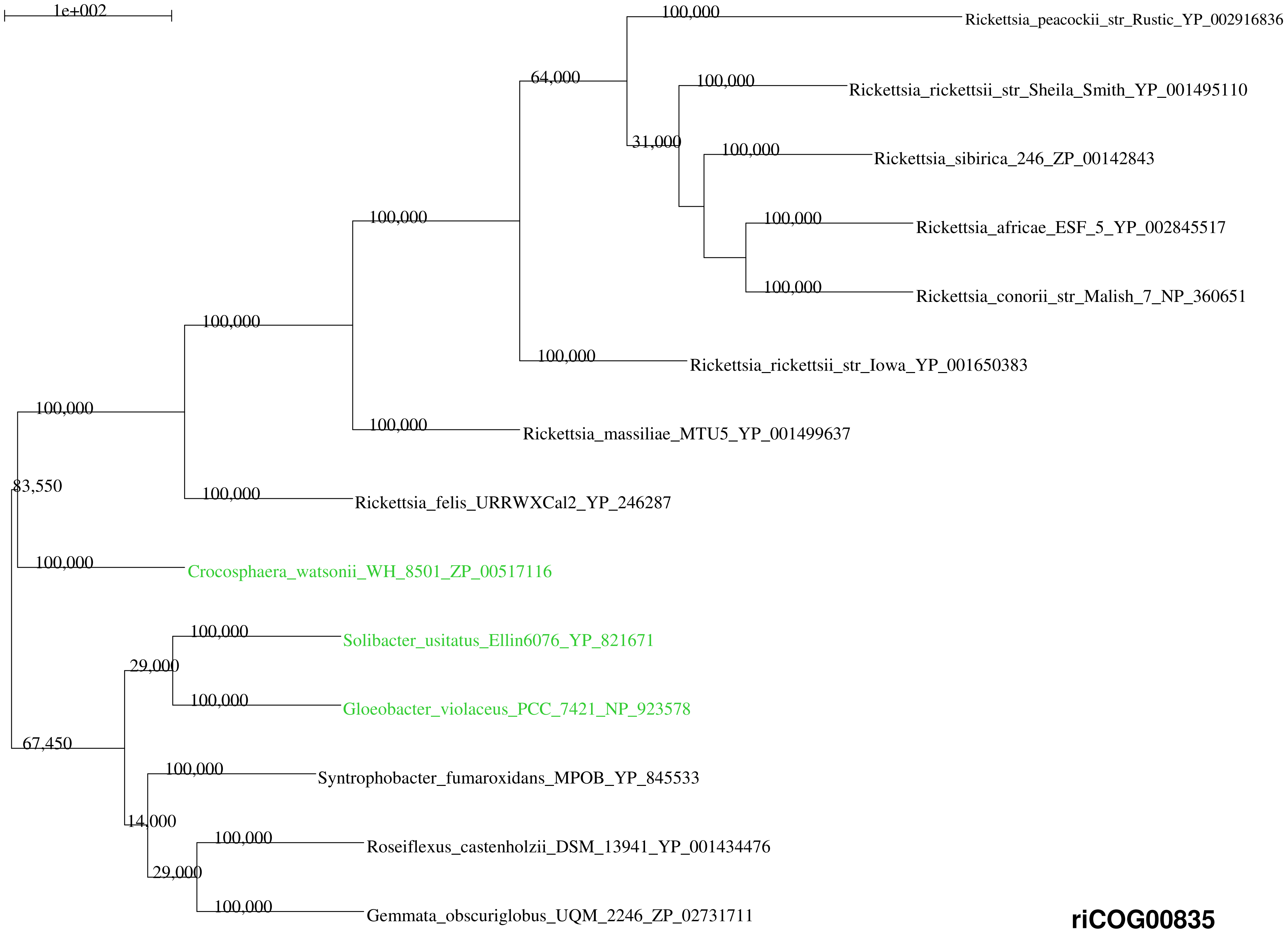
1e+002
100,000
Rickettsia_peacockii_str_Rustic_YP_002916836
64,000
100,000
Rickettsia_rickettsii_str_Sheila_Smith_YP_001495110
31,000
100,000
Rickettsia_sibirica_246_ZP_00142843
100,000
Rickettsia_africae_ESF_5_YP_002845517
100,000
Rickettsia_conorii_str_Malish_7_NP_360651
100,000
100,000
Rickettsia_rickettsii_str_Iowa_YP_001650383
100,000
100,000
Rickettsia_massiliae_MTU5_YP_001499637
100,000
83,550
100,000
Rickettsia_felis_URRWXCal2_YP_246287
100,000
Crocosphaera_watsonii_WH_8501_ZP_00517116
100,000
Solibacter_usitatus_Ellin6076_YP_821671
29,000
100,000
Gloeobacter_violaceus_PCC_7421_NP_923578
67,450
100,000
Syntrophobacter_fumaroxidans_MPOB_YP_845533
14,000
100,000
Roseiflexus_castenholzii_DSM_13941_YP_001434476
29,000
100,000
Gemmata_obscuriglobus_UQM_2246_ZP_02731711

riCOG00835

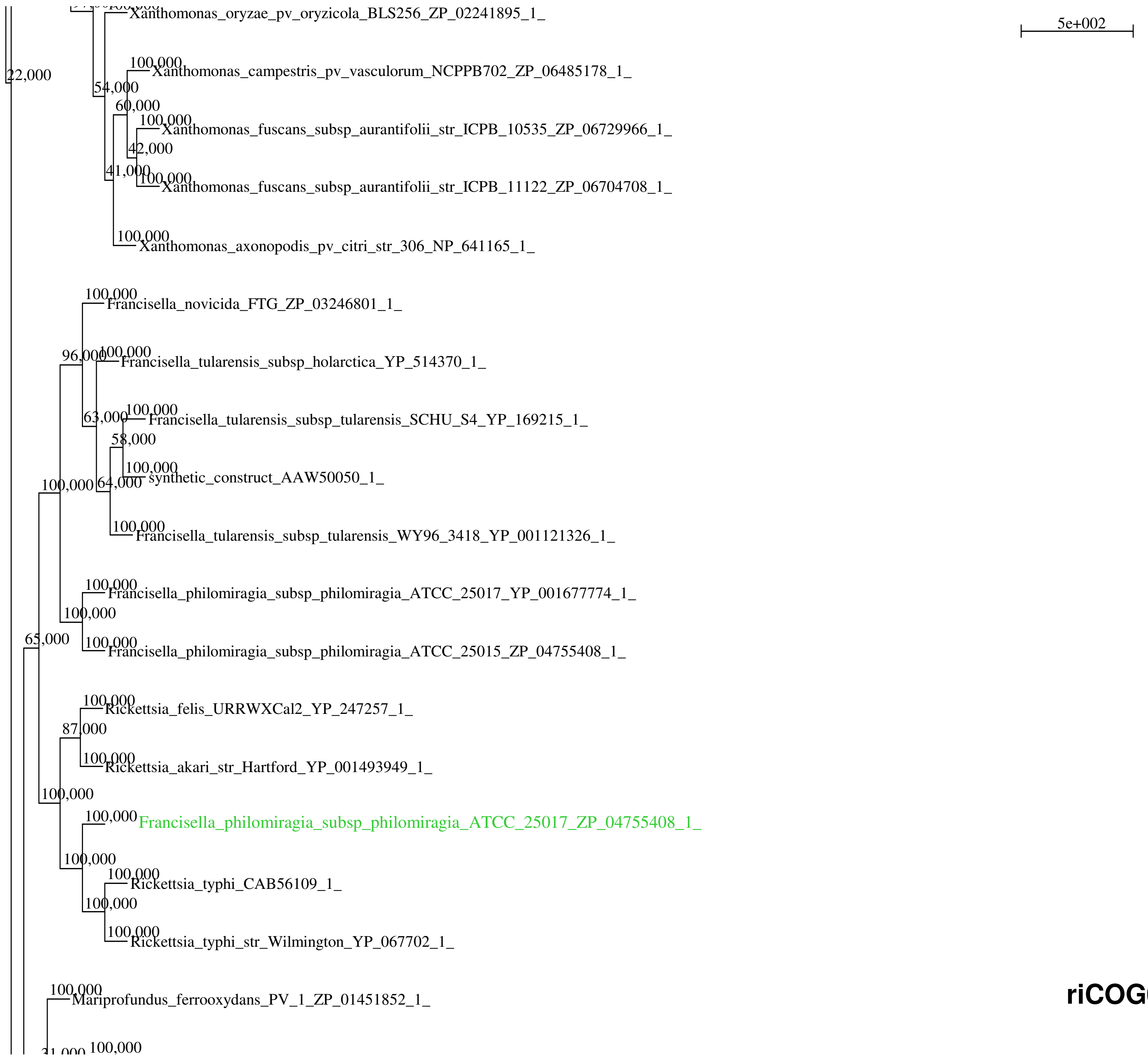
5e+002
Xanthomonas_oryzae_pv_oryzicola_BLS256_ZP_02241895_1_
22,000
100,000
Xanthomonas_campestris_pv_vasculorum_NCPPB702_ZP_06485178_1_
54,000
60,000
100,000
Xanthomonas_fuscans_subsp_aurantifolii_str_ICPB_10535_ZP_06729966_1_
42,000
41,000
100,000
Xanthomonas_fuscans_subsp_aurantifolii_str_ICPB_11122_ZP_06704708_1_
100,000
Xanthomonas_axonopodis_pv_citri_str_306_NP_641165_1_
100,000
Francisella_novicida_FTG_ZP_03246801_1_
96,000
100,000
Francisella_tularensis_subsp_holarctica_YP_514370_1_
63,000
100,000
Francisella_tularensis_subsp_tularensis_SCHU_S4_YP_169215_1_
58,000
100,000
synthetic_construct_AAW50050_1_
100,000
64,000
100,000
Francisella_tularensis_subsp_tularensis_WY96_3418_YP_001121326_1_
100,000
Francisella_philomiragia_subsp_philomiragia_ATCC_25017_YP_001677774_1_
100,000
65,000
100,000
Francisella_philomiragia_subsp_philomiragia_ATCC_25015_ZP_04755408_1_
100,000
Rickettsia_felis_URRWXCal2_YP_247257_1_
87,000
100,000
Rickettsia_akari_str_Hartford_YP_001493949_1_
100,000
100,000
Francisella_philomiragia_subsp_philomiragia_ATCC_25017_ZP_04755408_1_
100,000
100,000
Rickettsia_typhi_CAB56109_1_
100,000
100,000
Rickettsia_typhi_str_Wilmington_YP_067702_1_
100,000
Mariprofundus_ferrooxydans_PV_1_ZP_01451852_1_
100,000

riCOG00983

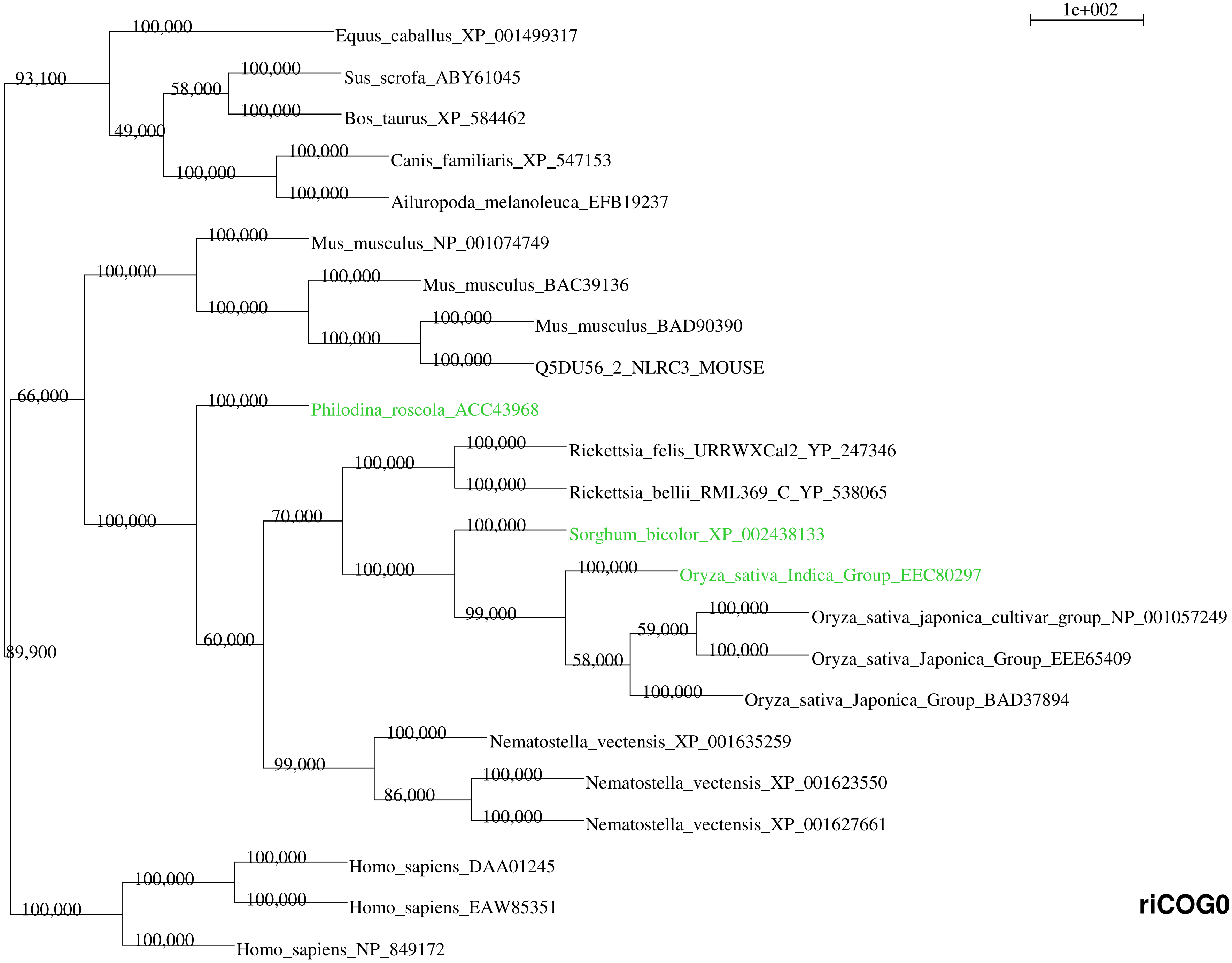
1e+002
Equus_caballus_XP_001499317
Sus_scrofa_ABY61045
Bos_taurus_XP_584462
Canis_familiaris_XP_547153
Ailuropoda_melanoleuca_EFB19237
Mus_musculus_NP_001074749
Mus_musculus_BAC39136
Mus_musculus_BAD90390
Q5DU56_2_NLRC3_MOUSE
Philodina_roseola_ACC43968
Rickettsia_felis_URRWXCal2_YP_247346
Rickettsia_bellii_RML369_C_YP_538065
Sorghum_bicolor_XP_002438133
Oryza_sativa_Indica_Group_EEC80297
Oryza_sativa_japonica_cultivar_group_NP_001057249
Oryza_sativa_Japonica_Group_EEE65409
Oryza_sativa_Japonica_Group_BAD37894
Nematostella_vectensis_XP_001635259
Nematostella_vectensis_XP_001623550
Nematostella_vectensis_XP_001627661
Homo_sapiens_DAA01245
Homo_sapiens_EAW85351
Homo_sapiens_NP_849172
93,100
49,000
58,000
66,000
89,900
70,000
60,000
99,000
59,000
86,000
100,000

riCOG01685

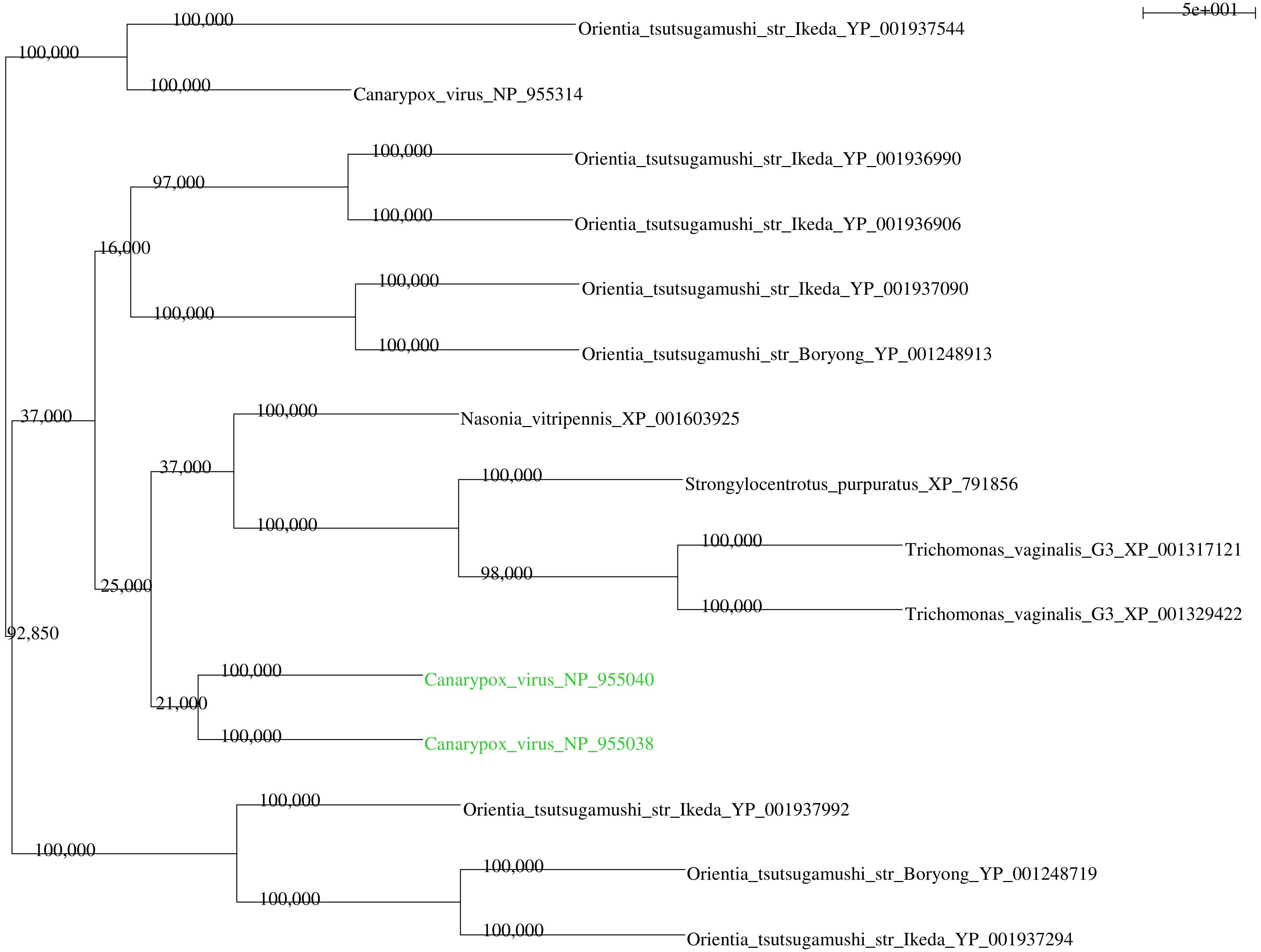

rioriCOG00831

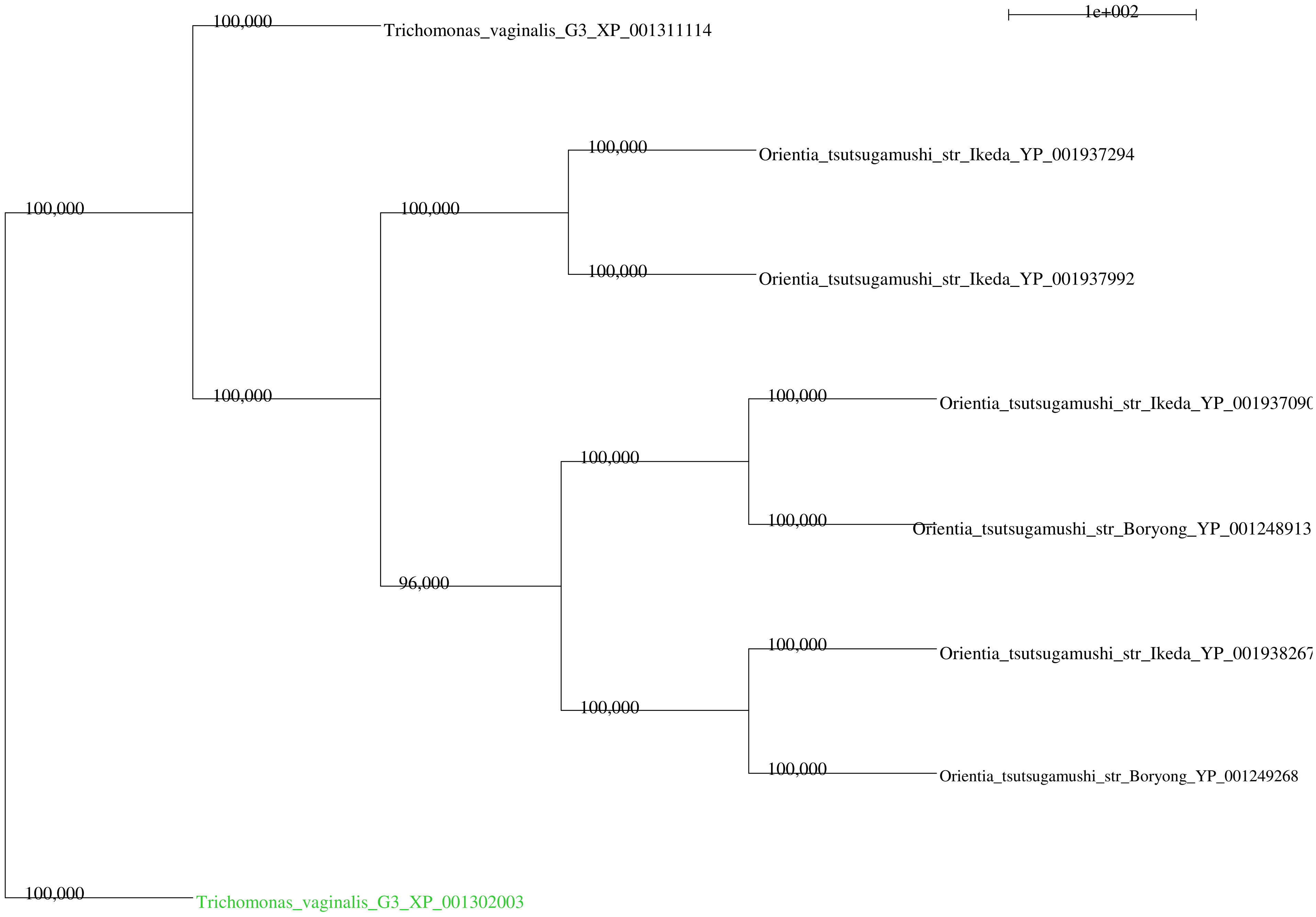

rioriCOG00862

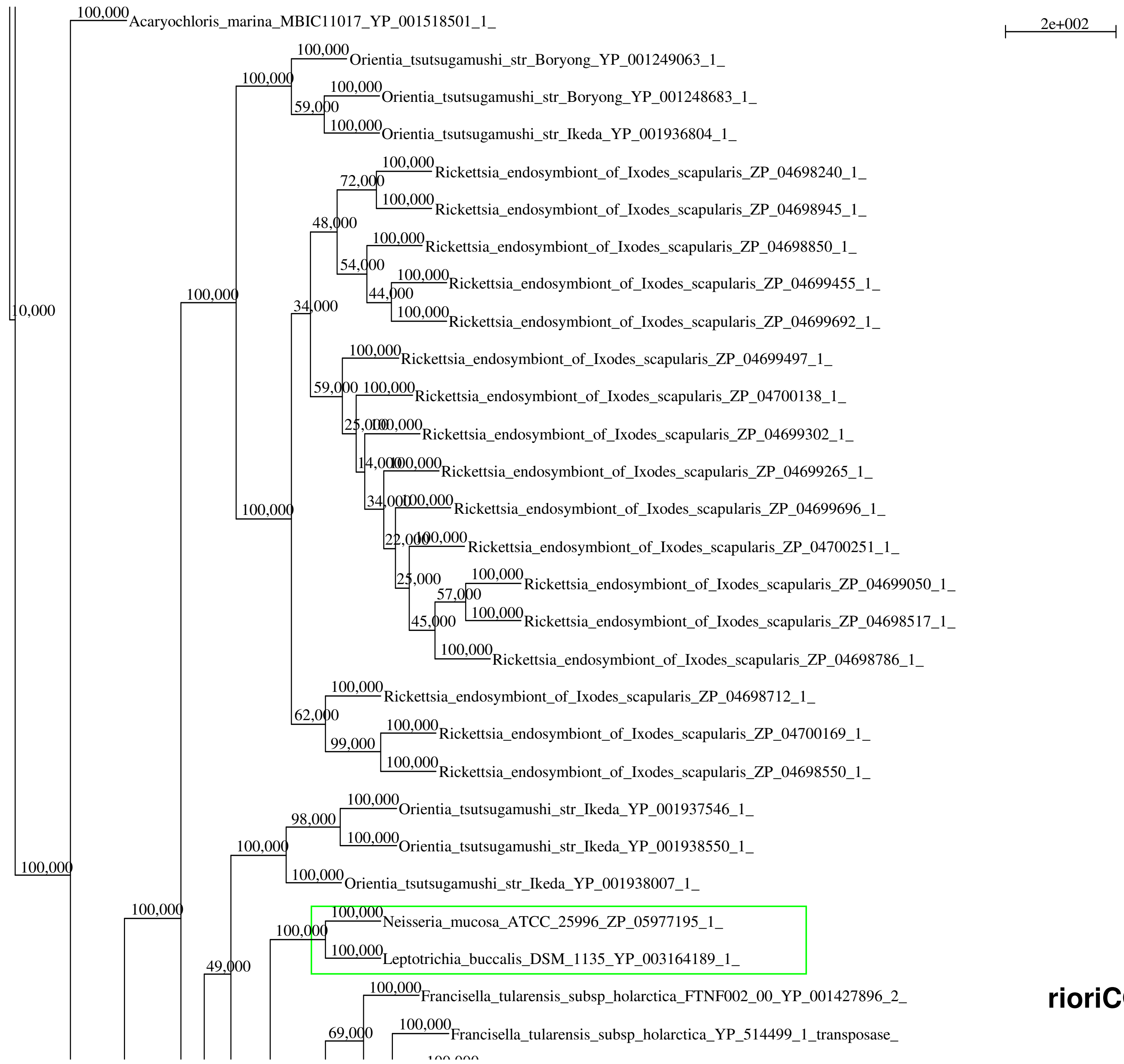

rioriCOG00900

Rickettsiales as gene donors

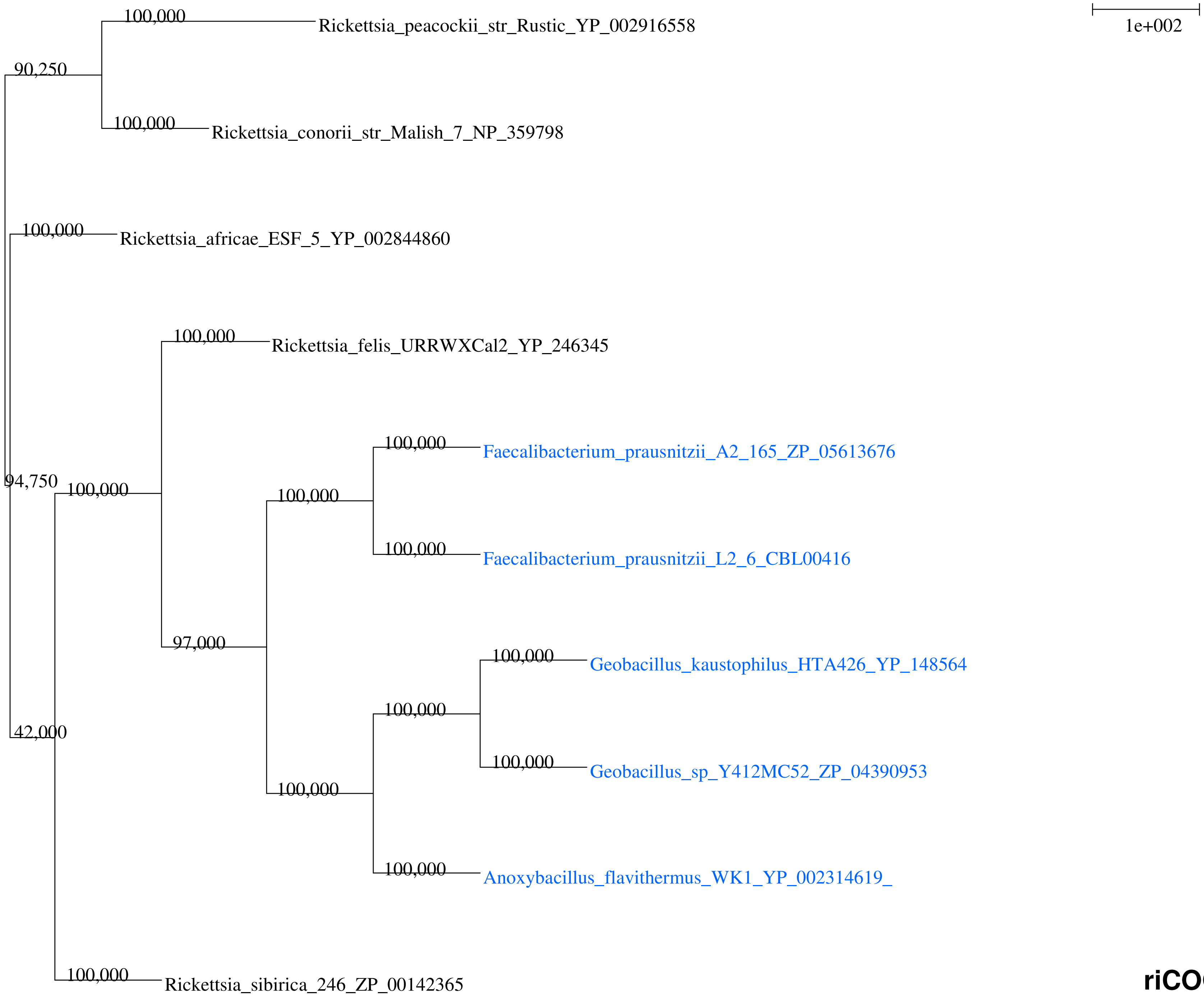
1e+002
100,000
Rickettsia_peacockii_str_Rustic_YP_002916558
90,250
100,000
Rickettsia_conorii_str_Malish_7_NP_359798
100,000
Rickettsia_africae_ESF_5_YP_002844860
100,000
Rickettsia_felis_URRWXCal2_YP_246345
100,000
Faecalibacterium_prausnitzii_A2_165_ZP_05613676
94,750
100,000
100,000
100,000
Faecalibacterium_prausnitzii_L2_6_CBL00416
97,000
100,000
Geobacillus_kaustophilus_HTA426_YP_148564
100,000
42,000
100,000
Geobacillus_sp_Y412MC52_ZP_04390953
100,000
100,000
Anoxybacillus_flavithermus_WK1_YP_002314619_
100,000
Rickettsia_sibirica_246_ZP_00142365

riCOG00139

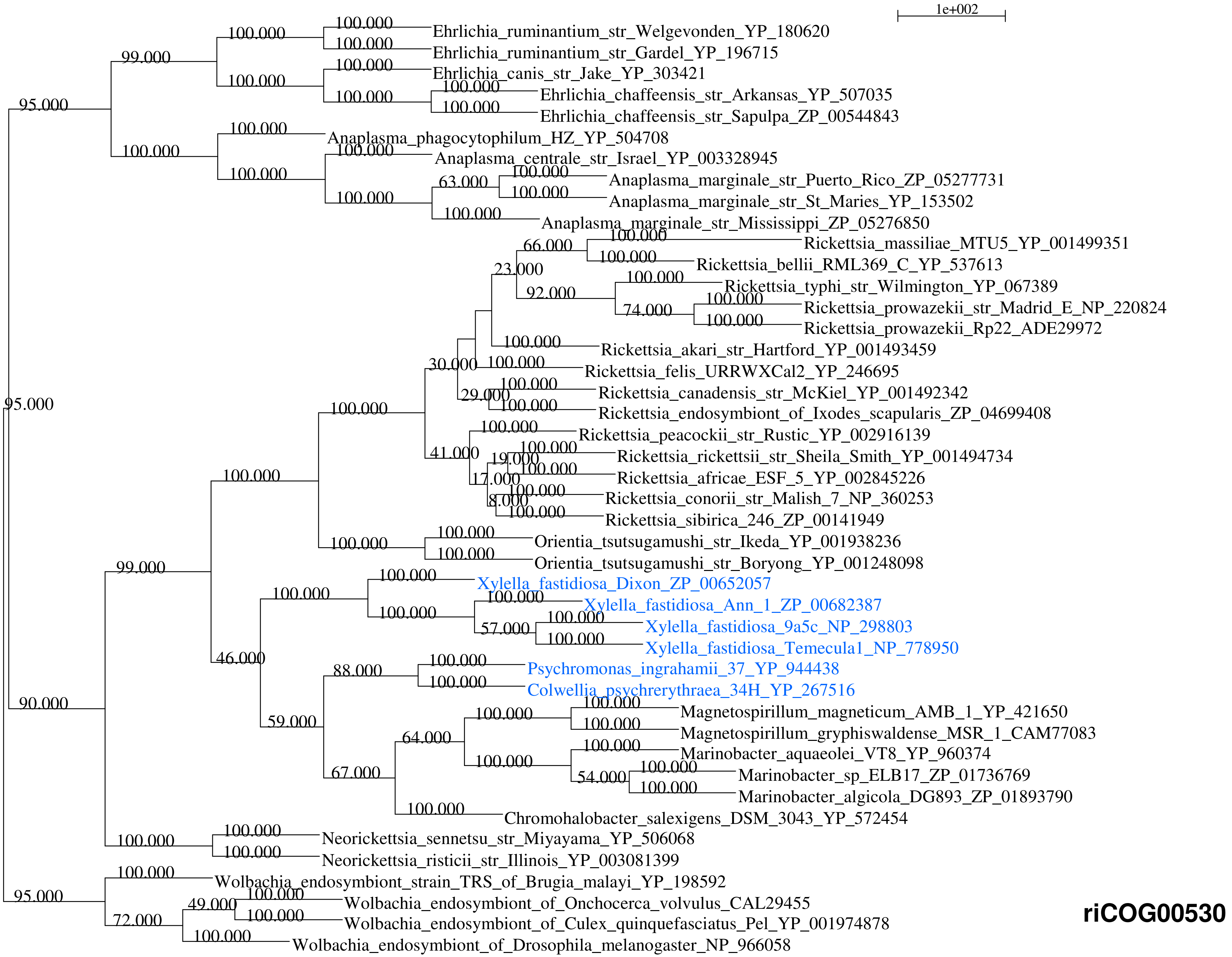
1e+002
Ehrlichia_ruminantium_str_Welgevonden_YP_180620
Ehrlichia_ruminantium_str_Gardel_YP_196715
Ehrlichia_canis_str_Jake_YP_303421
Ehrlichia_chaffeensis_str_Arkansas_YP_507035
Ehrlichia_chaffeensis_str_Sapulpa_ZP_00544843
Anaplasma_phagocytophilum_HZ_YP_504708
Anaplasma_centrale_str_Israel_YP_003328945
Anaplasma_marginale_str_Puerto_Rico_ZP_05277731
Anaplasma_marginale_str_St_Maries_YP_153502
Anaplasma_marginale_str_Mississippi_ZP_05276850
Rickettsia_massiliae_MTU5_YP_001499351
Rickettsia_bellii_RML369_C_YP_537613
Rickettsia_typhi_str_Wilmington_YP_067389
Rickettsia_prowazekii_str_Madrid_E_NP_220824
Rickettsia_prowazekii_Rp22_ADE29972
Rickettsia_akari_str_Hartford_YP_001493459
Rickettsia_felis_URRWXCal2_YP_246695
Rickettsia_canadensis_str_McKiel_YP_001492342
Rickettsia_endosymbiont_of_Ixodes_scapularis_ZP_04699408
Rickettsia_peacockii_str_Rustic_YP_002916139
Rickettsia_rickettsii_str_Sheila_Smith_YP_001494734
Rickettsia_africae_ESF_5_YP_002845226
Rickettsia_conorii_str_Malish_7_NP_360253
Rickettsia_sibirica_246_ZP_00141949
Orientia_tsutsugamushi_str_Ikeda_YP_001938236
Orientia_tsutsugamushi_str_Boryong_YP_001248098
Xylella_fastidiosa_Dixon_ZP_00652057
Xylella_fastidiosa_Ann_1_ZP_00682387
Xylella_fastidiosa_9a5c_NP_298803
Xylella_fastidiosa_Temecula1_NP_778950
Psychromonas_ingrahamii_37_YP_944438
Colwellia_psychrerythraea_34H_YP_267516
Magnetospirillum_magneticum_AMB_1_YP_421650
Magnetospirillum_gryphiswaldense_MSR_1_CAM77083
Marinobacter_aquaeolei_VT8_YP_960374
Marinobacter_sp_ELB17_ZP_01736769
Marinobacter_algicola_DG893_ZP_01893790
Chromohalobacter_salexigens_DSM_3043_YP_572454
Neorickettsia_sennetsu_str_Miyayama_YP_506068
Neorickettsia_risticii_str_Illinois_YP_003081399
Wolbachia_endosymbiont_strain_TRS_of_Brugia_malayi_YP_198592
Wolbachia_endosymbiont_of_Onchocerca_volvulus_CAL29455
Wolbachia_endosymbiont_of_Culex_quinquefasciatus_Pel_YP_001974878
Wolbachia_endosymbiont_of_Drosophila_melanogaster_NP_966058
riCOG00530

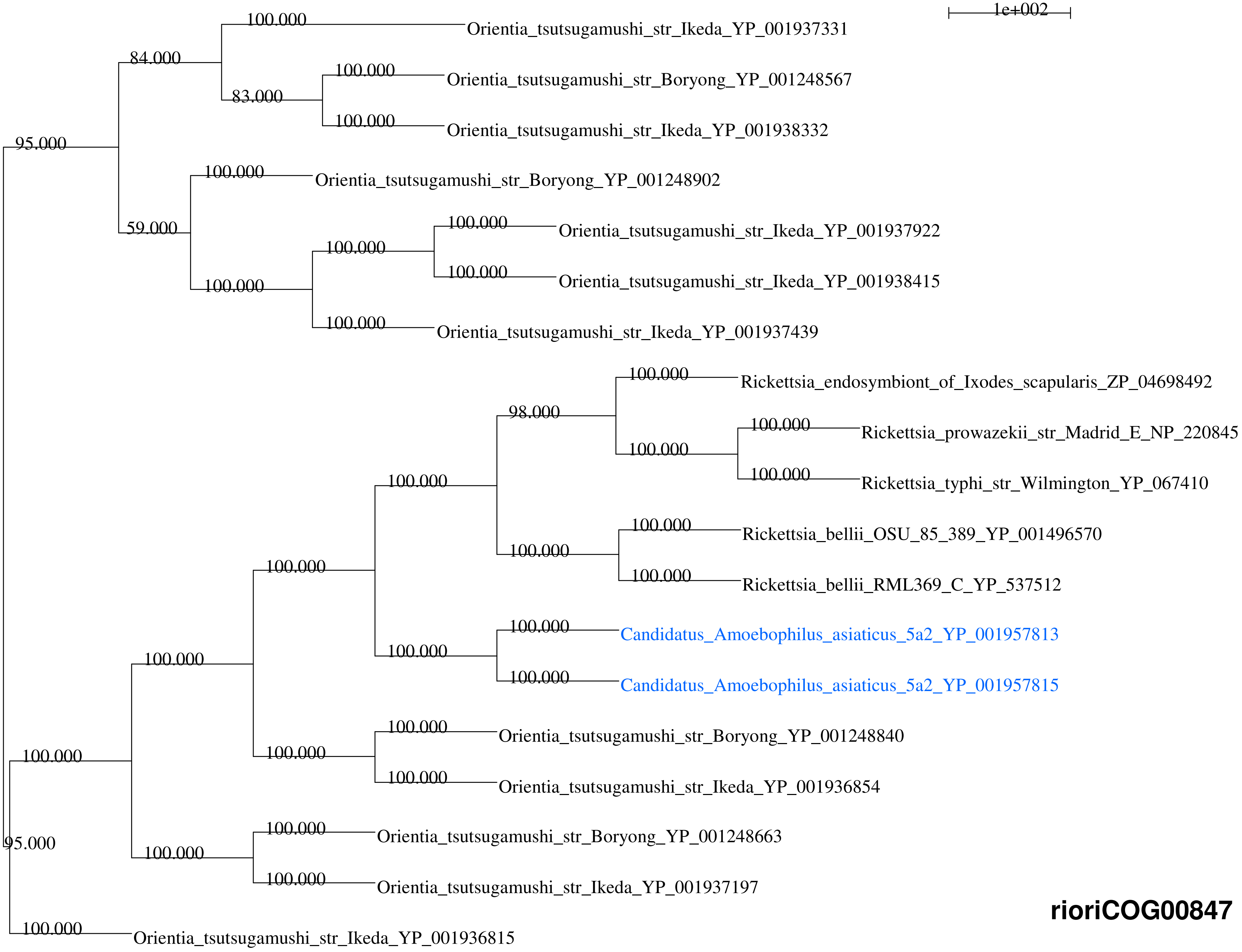

rioriCOG00847
